# Decoding Suicide Decedent Profiles and Signs of Suicidal Intent Using Latent Class Analysis

**DOI:** 10.1001/jamapsychiatry.2024.0171

**Published:** 2024-03-20

**Authors:** Yunyu Xiao, Kaiwen Bi, Paul Siu-Fai Yip, Julie Cerel, Timothy T. Brown, Yifan Peng, Jyotishman Pathak, J. John Mann

**Affiliations:** 1 Department of Population Health Sciences, Weill Cornell Medicine/NewYork-Presbyterian, New York; 2 Department of Social Work and Social Administration, The University of Hong Kong, Hong Kong; 3 Hong Kong Jockey Club Centre for Suicide Research and Prevention, The University of Hong Kong, Hong Kong; 4 College of Social Work, University of Kentucky, Lexington; 5 School of Public Health, University of California, Berkeley; 6 Department of Psychiatry, Columbia University Irving Medical Center, Columbia University, New York, New York; 7 Department of Radiology, Columbia University Irving Medical Center, Columbia University, New York, New York; 8 Division of Molecular Imaging and Neuropathology, New York State Psychiatric Institute, New York

## Abstract

**Importance:**

Suicide rates in the US increased by 35.6% from 2001 to 2021. Given that most individuals die on their first attempt, earlier detection and intervention are crucial. Understanding modifiable risk factors is key to effective prevention strategies.

**Objective:**

To identify distinct suicide profiles or classes, associated signs of suicidal intent, and patterns of modifiable risks for targeted prevention efforts.

**Design, Setting, and Participants:**

This cross-sectional study used data from the 2003-2020 National Violent Death Reporting System Restricted Access Database for 306 800 suicide decedents. Statistical analysis was performed from July 2022 to June 2023.

**Exposures:**

Suicide decedent profiles were determined using latent class analyses of available data on suicide circumstances, toxicology, and methods.

**Main Outcomes and Measures:**

Disclosure of recent intent, suicide note presence, and known psychotropic usage.

**Results:**

Among 306 800 suicide decedents (mean [SD] age, 46.3 [18.4] years; 239 627 males [78.1%] and 67 108 females [21.9%]), 5 profiles or classes were identified. The largest class, class 4 (97 175 [31.7%]), predominantly faced physical health challenges, followed by polysubstance problems in class 5 (58 803 [19.2%]), and crisis, alcohol-related, and intimate partner problems in class 3 (55 367 [18.0%]), mental health problems (class 2, 53 928 [17.6%]), and comorbid mental health and substance use disorders (class 1, 41 527 [13.5%]). Class 4 had the lowest rates of disclosing suicidal intent (13 952 [14.4%]) and leaving a suicide note (24 351 [25.1%]). Adjusting for covariates, compared with class 1, class 4 had the highest odds of not disclosing suicide intent (odds ratio [OR], 2.58; 95% CI, 2.51-2.66) and not leaving a suicide note (OR, 1.45; 95% CI, 1.41-1.49). Class 4 also had the lowest rates of all known psychiatric illnesses and psychotropic medications among all suicide profiles. Class 4 had more older adults (23 794 were aged 55-70 years [24.5%]; 20 100 aged ≥71 years [20.7%]), veterans (22 220 [22.9%]), widows (8633 [8.9%]), individuals with less than high school education (15 690 [16.1%]), and rural residents (23 966 [24.7%]).

**Conclusions and Relevance:**

This study identified 5 distinct suicide profiles, highlighting a need for tailored prevention strategies. Improving the detection and treatment of coexisting mental health conditions, substance and alcohol use disorders, and physical illnesses is paramount. The implementation of means restriction strategies plays a vital role in reducing suicide risks across most of the profiles, reinforcing the need for a multifaceted approach to suicide prevention.

## Key Points

**Question:**

Are there distinct suicide profiles linked to varying signs of suicidal intent and risks?

**Findings:**

This cross-sectional study including 306 800 US suicide decedents from 2003-2020 identified 5 suicide profiles or classes: comorbid mental health and substance use disorders; mental disorders alone; crisis, alcohol-related, and intimate partner problems; physical health problems; and polysubstance use. Class 4 (physical health problems) was the largest, exhibiting minimal explicit suicidal intent, rarely detected psychiatric diagnoses, and minimal psychotropic medication use.

**Meaning:**

Suicide prevention strategies may be more effective when tailored to different suicide profiles because integrated care enhances the detection and treatment of comorbid mental health conditions, substance and alcohol use disorders, and physical health problems.

## Teaser

This cross-sectional study examines data from the 2003-2020 National Violent Death Reporting System to identify distinct suicide profiles among US suicide decedents and determine associations to signs of suicidal intent and risks.

## Introduction

Suicide rates in the United States increased by 35.6% from 10.4 to 14.1 per 100 000 per year between 2001 and 2021.^1^ This increase is particularly concerning given that up to 79% of suicide decedents die on their first attempt,^2,3,4^ underscoring the need for effective early detection and intervention.^5^

The multifaceted nature of suicide, as evidenced by the absence of a single profile,^5,6^ challenges traditional prevention efforts that predominantly focus on screening known mental disorders.^7^ Although psychological autopsy studies indicate 70% to 90% of suicides involved a mental disorder,^8,9,10,11,12^ only about 20% of suicide decedents with a psychiatric disorder before death had engaged with mental health services.^13^ This suggests a gap in prevention strategies: identifying individuals with mental disorders in mental health settings is insufficient,^14^ as that approach fails to reach most of those at risk. Among varying lethality of methods, self-inflicted gunshots have a survival rate of less than 10% compared with a 92% survival rate for drug overdoses, which adds complexity.^15^ Suicide decedents using high-lethality methods were less likely to seek psychiatric treatment or have a history of previous nonfatal suicide attempts.^16^ Thus, suicide prevention in this high-risk subgroup must begin before the first suicide attempt and outside mental health service settings.

To improve prevention, a better understanding of modifiable risk factors is imperative.^2,5^ One approach is identifying “suicide decedent profiles” with a broader spectrum of risk factors: seeking at-risk subgroups that may not manifest traditional warning signs,^17^ such as individuals with diagnosed mental health disorders, psychiatric medications, nonfatal suicide attempts, or disclosure of suicidal intent.^17,18^ Instead of a 1-size-fits-all approach, identifying heterogeneous suicide decedent profiles may inform prevention strategies tailored for high-risk populations who might die at their first attempt.^19^

Previous efforts using this approach have encountered limitations. First, our study, leveraging the National Violent Death Record System (NVDRS), addresses a critical gap in suicide research by focusing on data typically absent in clinical settings. This approach is necessary and innovative, because within 1 month of death, twice as many individuals (40%) had consulted general health care professionals^20^ compared with mental health professionals.^13^ Second, while it is labor intensive to use psychological autopsies to detect mental disorders, we used toxicology data from medical/legal examiners to assess psychotropic treatment adherence in suicide decedents.^2,21^ This information, coupled with suicide method details from autopsy records, offers a more comprehensive understanding of suicide decedent profiles than previous studies.^22,23,24^ Third, most US studies lacked national representation, with previous research using NVDRS datasets limited to 17 states.^24^ Fourth, despite evidence of disparities in suicide deaths and precipitating circumstances across age, race and ethnicity, sex, and geography,^6,22^ mapping these characteristics onto suicide profiles remains underexplored.

Our study addresses these gaps by discerning suicide profiles through a comprehensive analysis of variables, including circumstances of death, toxicology, and suicide methods. We analyzed all suicides from 2003 to 2020 in the NVDRS Restricted Access Database. Additionally, we associated suicides with suicide intent disclosure, suicide notes, and psychotropic use while examining sociodemographic differences. Our data-driven approach led us to hypothesize distinct suicide profiles that differ regarding potentially modifiable risk factors and would require different combinations of evidence-based prevention measures in nonpsychiatric and psychiatric clinical settings.

## Methods

### Study Design, Setting, and Population

The NVDRS is a national surveillance system for violent deaths,^25^ the largest dataset on US suicide decedents. Beginning in 2003 in 6 states and expanding to 50 states, the District of Columbia, and Puerto Rico by 2018,^26^ the NVDRS Restricted Access Database compiles information from death certificates, coroner and medical examiner records, and toxicology reports. Abstractors review data to ensure accuracy.^27^ The system is suitable for identifying suicide profiles (eMethods 1 in Supplement 1). We included all 306 800 suicide decedents at all ages from 2003 to 2020. Statistical analyses were performed from July 2022 to June 2023. Weill Cornell Medicine’s institutional review board exempted this study. We followed Strengthening the Reporting of Observational Studies in Epidemiology (STROBE) reporting guidelines for cross-sectional designs.

### Measures

Latent class analysis models included theory-informed^28,29,30,31^ and evidence-informed^32,33^ indicators: circumstances, toxicology-based substance indicators, and suicide-method indicators (see eTable 1 for data dictionary and recoding and eMethods 2 for indicator selection rationale in Supplement 1).

Circumstances included mental health problems, depressed mood, past/current mental illness treatment, suicide-attempt history, recent crisis, financial problems, job-related problems, friend/family member suicide/death, physical health problems, interpersonal violence perpetration/victimhood, criminal/civil legal problems, intimate partner/other relationship problems, alcohol, and other substance-use problems. Responses were binary (0 = no/not available/unknown; 1 = yes). See eMethods 3 and 4 in Supplement 1 for a discussion of no/not available/unknown responses and other recoding.

Toxicology screening included amphetamines, alcohol, opiates, marijuana, and cocaine. We recoded responses into binary variables (0 = no/not available/unknown/missing; 1 = yes) after assessing missing/misclassification (eMethods 4 in Supplement 1). We calculated polysubstance use (0 = none present/unknown/missing, 1 = 1, 2 = 2, 3 = 3, 4 = ≥4 substances).

We grouped suicide methods into 4 categories: 0 indicated firearms; 1, hanging, suffocation, or strangulation; 2, poisoning (suicide by prescription/illicit drugs, alcohol, carbon monoxide, gas, rat poison, or insecticides); and 3, other methods (eg, fire, drowning, hypothermia) or unknown/missing.^13^

Primary outcomes were the absence of suicide intent disclosure and suicide notes. Suicide intent disclosure indicated disclosing suicidal thoughts or plans within the last month to family members, health care workers, friends, intimate partners, neighbors, others, or on social media. Suicide note presence means the decedent left a suicide note or other communication indicating intent but too late for prevention. Additionally, we evaluated the presence of 4 psychotropic drug classes (antidepressant, anticonvulsant, antipsychotic, and benzodiazepine) from toxicology reports. Because not every drug was tested, a decision made by medical/legal examiners, we calculated new variables accounting for whether the drug was tested and the results (response categories: tested–present, tested–not present, not tested, and missing or unknown). We assessed missing values (eTable 1 in Supplement 1).

We included suicide-associated covariates: age, sex, race and ethnicity, education, marital status, military/veteran status, and urban/rural residence.^5,6,34,35^ Race and ethnicity descriptions included Hispanic, non-Hispanic American Indian or Alaska Native (hereafter American Indian or Alaska Native), non-Hispanic Asian or Other Pacific Islander (hereafter Asian or Other Pacific Islander), non-Hispanic Black (hereafter Black), non-Hispanic other/unspecified, non-Hispanic 2 or more races, non-Hispanic White (hereafter White), and unknown/missing.^36^

### Statistical Analysis

We conducted latent class analysis using poLCA^37^ in R version 4.2.1 (R Foundation) to identify profiles. Latent class analysis is a finite mixture modeling approach to identify groups based on similar indicator responses.^23,38,39^ We estimated models ranging from 2 to 10 classes, determining the optimal model by fit (eg, Bayesian information criterion, entropy) and interpretability.

Individuals were assigned to classes based on probability,^40^ with class prevalence and membership differences analyzed using analysis of variance, Kruskal-Wallis, or χ^2^ tests. Logistic regression was used to associate class membership with primary outcomes.

There were no missing values for class indicators and outcome variables after recoding. We addressed missing covariate data (0.0%-19.3%) using multiple imputation (mice version 3.14.0)^41^ and validated findings against nonimputed data for robustness.

## Results

Among 306 800 suicide decedents, 239 627 (78.1%) were male, 67 108 were female (21.9%), 3918 (1.3%) were American Indian or Alaska Native, 7019 (2.3%) were Asian or Other Pacific Islander, 19 986 (6.5%) were Black, 19 354 (6.3%) were Hispanic, 251 861 (82.1%) were White, and 3428 (1.1%) reported more than 2 races (Table 1). There were 51 956 (16.9%) veterans. Regarding marital status, 109 151 (35.6%) were never married, and 99 532 (32.4%) were married or in a civil union or domestic partnership. Most completed at least high school (204 432 [66.6%]) and lived in metropolitan/urban areas (245 042 [79.9%]).

**Table 1.** Demographic Characteristics^a^

Characteristic	No. (%)					
	Overall (N = 306 800)	Class 1: mental health and substance problems (n = 41 527 [13.5%])	Class 2: mental health problems (n = 53 928 [17.6%])	Class 3: crisis, alcohol-related, and intimate partner problems (n = 55 367 [18.0%])	Class 4: physical health problems (n = 97 175 [31.7%])	Class 5: polysubstance problems (n = 58 803 [19.2%])
Age, y						
≤10	161 (0.1)	10 (0.0)	28 (0.1)	26 (0.0)	93 (0.1)	6 (0.0)
11-15	4926 (1.6)	447 (1.1)	1144 (2.1)	781 (1.4)	2125 (2.2)	429 (0.7)
16-24	37 319 (12.2)	4144 (10.0)	6796 (12.6)	8328 (15.0)	11 345 (11.7)	6706 (11.4)
25-40	80 996 (26.4)	12 031 (29.0)	13 543 (25.1)	19 014 (34.3)	19 238 (19.8)	17 170 (29.2)
41-54	81 037 (26.4)	13 454 (32.4)	14 977 (27.8)	16 431 (29.7)	20 314 (20.9)	15 861 (27.0)
55-70	68 290 (22.3)	9526 (22.9)	12 535 (23.2)	9045 (16.3)	23 794 (24.5)	13 390 (22.8)
≥71	33 894 (11.0)	1915 (4.6)	4905 (9.1)	1740 (3.1)	20 100 (20.7)	5234 (8.9)
Unknown/missing	175 (0.1)	0	0	2 (0.0)	166 (0.2)	7 (0.0)
Sex						
Male	239 627 (78.1)	23 962 (57.7)	40 703 (75.5)	49 019 (88.5)	82 191 (84.6)	43 752 (74.4)
Female	67 108 (21.9)	17 563 (42.3)	13 225 (24.5)	6347 (11.5)	14 923 (15.4)	15 050 (25.6)
Unknown/missing	65 (0.0)	2 (0.0)	0	1 (0.0)	61 (0.1)	1 (0.0)
Race and ethnicity						
American Indian or Alaska Native^b^	3918 (1.3)	415 (1.0)	389 (0.7)	996 (1.8)	1238 (1.3)	880 (1.5)
Asian or Other Pacific Islander^b^	7019 (2.3)	686 (1.7)	1178 (2.2)	1187 (2.1)	2541 (2.6)	1427 (2.4)
Black or African American^b^	19 986 (6.5)	1682 (4.1)	2764 (5.1)	4422 (8.0)	7014 (7.2)	4104 (7.0)
Hispanic	19 354 (6.3)	2220 (5.3)	2545 (4.7)	4584 (8.3)	5253 (5.4)	4752 (8.1)
Other/unspecified^b^	912 (0.3)	68 (0.2)	79 (0.1)	99 (0.2)	491 (0.5)	175 (0.3)
≥2 Races^b^	3428 (1.1)	432 (1.0)	625 (1.2)	826 (1.5)	923 (0.9)	622 (1.1)
White^b^	251 861 (82.1)	36 010 (86.7)	46 333 (85.9)	43 225 (78.1)	79 485 (81.8)	46 808 (79.6)
Unknown/missing	322 (0.1)	14 (0.0)	15 (0.0)	28 (0.1)	230 (0.2)	35 (0.1)
Military affiliation						
Yes	51 956 (16.9)	4301 (10.4)	9113 (16.9)	8563 (15.5)	22 220 (22.9)	7759 (13.2)
No	238 413 (77.7)	35 298 (85.0)	42 310 (78.5)	44 324 (80.1)	68 568 (70.6)	47 913 (81.5)
Unknown/missing	16 431 (5.4)	1928 (4.6)	2505 (4.6)	2480 (4.5)	6387 (6.6)	3131 (5.3)
Marital status						
Married, civil union, domestic partnership	99 532 (32.4)	12 152 (29.3)	19416 (36.0)	19 144 (34.6)	32 609 (33.6)	16 211 (27.6)
Divorced	65 066 (21.2)	10 273 (24.7)	10 571 (19.6)	11 040 (19.9)	19 832 (20.4)	13 350 (22.7)
Married, civil union, domestic partnership but separated	7531 (2.5)	1419 (3.4)	1536 (2.8)	2383 (4.3)	982 (1.0)	1211 (2.1)
Never married	109 151 (35.6)	15 034 (36.2)	18 916 (35.1)	20 006 (36.1)	32 248 (33.2)	22 947 (39.0)
Single, not otherwise specified	3968 (1.3)	437 (1.1)	668 (1.2)	940 (1.7)	1075 (1.1)	848 (1.4)
Widowed	17 869 (5.8)	1864 (4.5)	2479 (4.6)	1447 (2.6)	8633 (8.9)	3446 (5.9)
Unknown/missing	3683 (1.2)	348 (0.8)	342 (0.6)	407 (0.7)	1796 (1.8)	790 (1.3)
Education level						
High school or above	204 432 (66.6)	29 853 (71.9)	34 165 (63.4)	34 300 (62.0)	62 898 (64.7)	43 216 (73.5)
Below high school	43 044 (14.0)	4486 (10.8)	6363 (11.8)	7774 (14.0)	15 690 (16.1)	8732 (14.8)
Unknown/missing	59 324 (19.3)	7188 (17.3)	13 401 (24.8)	13 293 (24.0)	18 587 (19.1)	6855 (11.7)
Residence^c^						
Nonmetropolitan	60 143 (19.6)	4905 (13.7)	9511 (17.6)	11382 (20.6)	23 966 (24.7)	9600 (16.3)
Metropolitan	245 042 (79.9)	30 755 (86.0)	44 253 (82.1)	43 792 (79.1)	72 421 (74.5)	48 855 (83.1)
Unknown/missing	1615 (0.5)	94 (0.3)	164 (0.3)	193 (0.3)	788 (0.8)	348 (0.6)

^a^ For all groups, *P* < .001 and adjusted *P* < .001. Test statistic and *P* value for Kruskal-Wallis tests or χ^2^ tests were used to compare all suicide deaths associated with latent classes of profiles (class 1 vs class 2 vs class 3 vs class 4 vs class 5). Bonferroni-adjusted *P* < .00625 (0.05/8 tests). Unknown/missing values were excluded when conducting the Kruskal-Wallis test or Pearson χ^2^ test.

^b^ Non-Hispanic ethnicity.

^c^ A metropolitan area was defined as scoring 3 or less on the Rural-Urban Continuum Code and a nonmetropolitan area, 4 or more.^35^

### Latent Classes of Suicide Decedents

**Figure 1.**
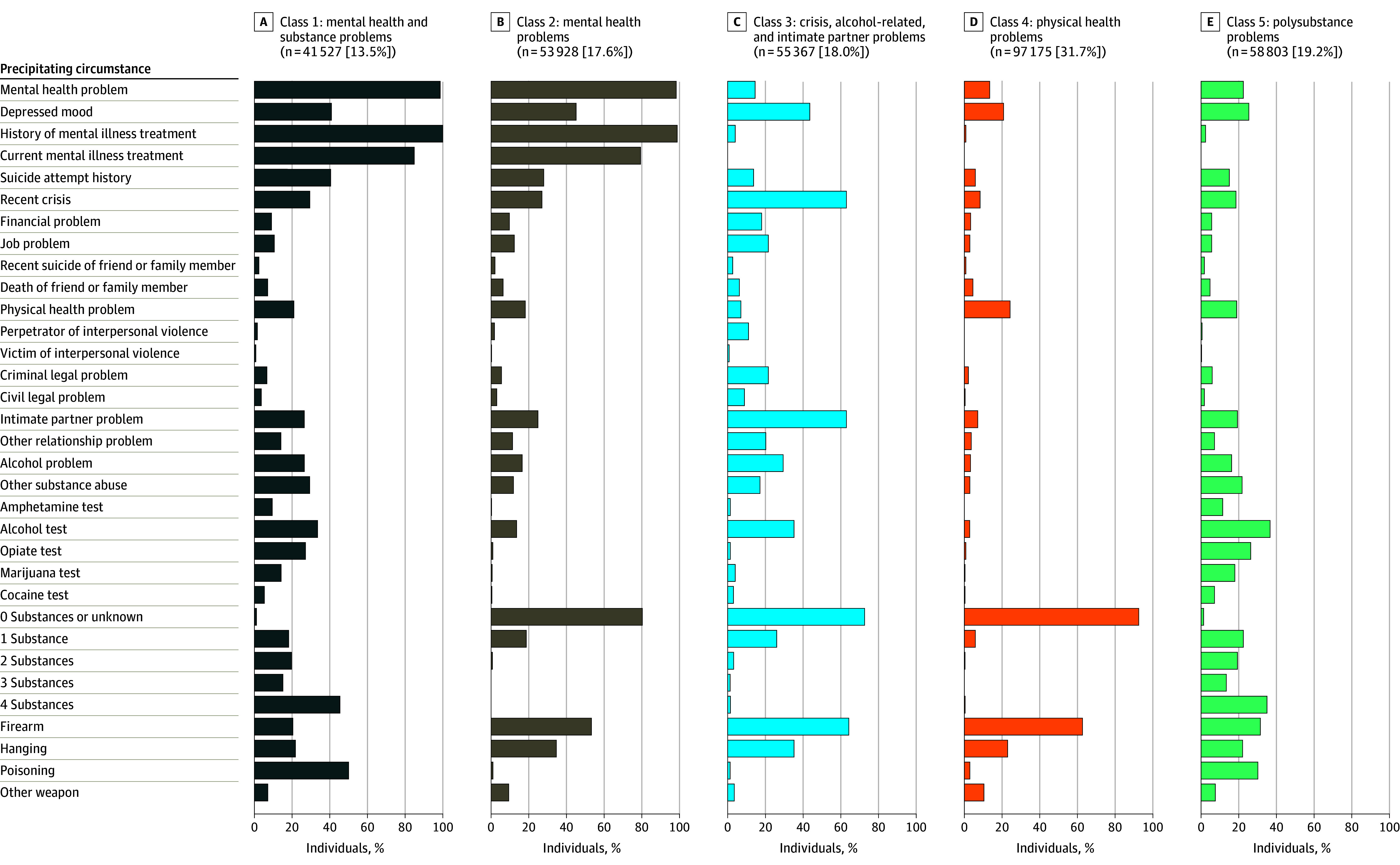
Distribution of Precipitating Circumstances by 5 Suicide Decedent Profiles in the US, 2003-2020 (N = 306 800)

A 5-class model (Figure 1) provided the best fit (eTable 2 and eFigure 1 in Supplement 1) and interpretability (eTable 3 and eFigures 2-10 in Supplement 1). Class 1 (mental health and substance problems, n = 41 527; 13.5%) had high percentages of mental health problems (40 979 [98.7%]), depressed mood (16 981 [40.9%]), past (41 409 [99.7%]) and current (35 217 [84.8%]) mental health treatment, and prior suicide attempts (16 811 [40.5%]). Almost everyone (41 031 [98.8%]) in class 1 had illegal substances detected on toxicology, with 45.8% (n = 19 029) of these decedents exhibiting 4 or more substances. Class 1 had the highest suicide proportion by poisoning (20 829 [50.2%]).

Class 2 (mental health problems, 53 928 [17.6%]) had comparably high known prior mental health problems (53 305 [98.8%]) but fewer substance problems than class 1 (80.5% [43 390] of class 2 had no substances at death, and <1% [427] had ≥2 substances). Class 2 had the highest proportion of suicides by hanging (18 887 [35.0%]). Class 3 (crisis, alcohol-related, and intimate-partner problems, 55 367 [18.0%]) manifested the most recent crisis, alcohol-related, and/or intimate partner problems.

Class 4 (physical health problems, 97 175 [31.7%]) was the largest class of suicide decedents and was characterized by the most physical health problems (23 647 [24.3%]) and firearm suicide deaths (61 674 [63.5%]). Class 4 had the fewest known mental health problems (13 033 [13.4%]), depressed mood (20 710 [21.3%]), positive amphetamine test results (44 [0.05%]), positive alcohol test results (2755 [2.8%]), positive opiate test results (717 [0.7%]), positive marijuana test results (229 [0.2%]), positive cocaine test results (67 [0.1%]), and prior suicide attempts (5561 [5.7%]), and less than 1% had known history of (668 [0.7%]) or current (10 [0.01%]) mental illness treatment.

Class 5 (polysubstance problems, n = 58 803, 19.2%) reported the most substance misuse (43 578 [74.1%] with ≥2 substances at death) and alcohol misuse (23 298 [39.6%]). Classes 1 and 5 differed: class 1 had a higher frequency of known mental health issues and treatment (40 979 [98.7%] and 41 409 [99.7%], respectively) compared with lower rates in class 5 (14 421 [24.5%] and 1697 [2.9%], respectively). Class 1 primarily attempted suicide by poisoning (20 829 [50.2%]), while class 5 mostly used firearms (20 220 [34.4%]).

**Figure 2.**
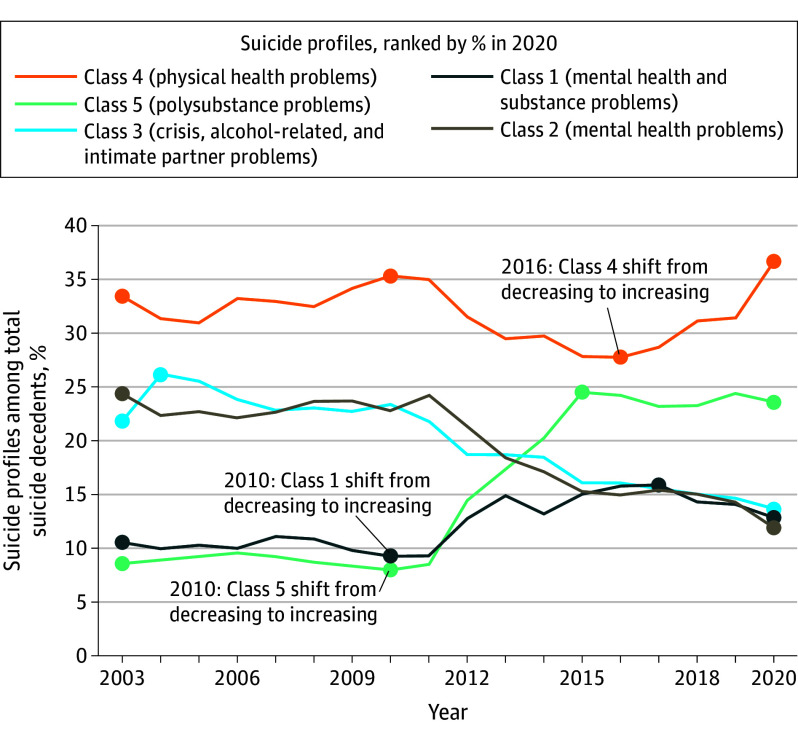
Temporal Changes of Identified Latent Classes of Suicide Decedent Profiles in the US, 2003-2020

Shifts in class proportions were observed (Figure 2). Class 5 saw the largest increase, rising by 194% from 2010 to 2015 ([8.4%] to 5075 [24.7%]). Class 4 increased by 31.2% in 2016 to 2020 (7223 [27.9%] to 14 198 [36.6%]). In contrast, class 2 and class 3 decreased 49.8% (888 [24.5%] to 4769 [12.3%], 2003-2020) and 36.7% (800 [22.1%] to 5436 [14.0%], 2003-2020), respectively. Between 2011 and 2017, class 1 increased by 67.0%, from 1206 (9.7%) to 4821 (16.2%).

### Associations With Signs of Suicide Intent

Known suicide intent disclosure and not leaving a suicide note varied across classes (Table 2). Class 1 (12 217 [29.4%]) and class 2 (15 708 [29.1%]) showed the highest rates of suicide intent disclosure, whereas class 4 (13 952 [14.4%]) had the lowest. Suicide notes ranged from the highest in class 1 (14 706 [35.4%]) and class 5 (18 445 [31.4%]) to the lowest in class 4 (24 351 [25.1%]). Psychotropic drugs detected via toxicology also varied, with class 1 showing the highest rates for antidepressants (17 551 [50.5%]), anticonvulsants (4366 [19.1%]), antipsychotics (3693 [16.2%]), and benzodiazepines (10 930 [41.5%]). In contrast, class 4 had the lowest rates of psychotropics detected: antidepressants (824 [2.3%]), antipsychotics (39 [0.3%]), and benzodiazepines (534 [3.5%]). Adjusting for covariates (Table 3), compared with class 1, the odds of not disclosing suicide intent were highest in class 4 (OR, 2.58; 95% CI, 2.51-2.66), and suicide note absence was more common in class 4 (OR, 1.45; 95% CI, 1.41-1.49).

**Table 2.** Distribution of Signs of Suicide Intent by 5 Suicide Decedent Profiles

Characteristic	No. (%)				
	Class 1: mental health and substance problems (n = 41 527 [13.5%])	Class 2: mental health problems (n = 53 928 [17.6%])	Class 3: crisis, alcohol-related, and intimate partner problems (n = 55 367 [18.0%])	Class 4: physical health problems (n = 97 175 [31.7%])	Class 5: polysubstance problems (n = 58 803 [19.2%])
Recent suicide disclosure^a^					
No	29 310 (70.6)	38 220 (70.9)	40 138 (72.5)	83 223 (85.6)	46 626 (79.3)
Yes	12 217 (29.4)	15 708 (29.1)	15 229 (27.5)	13 952 (14.4)	12 177 (20.7)
Suicide note^a^					
No	26 821 (64.6)	37 329 (69.2)	39 228 (70.9)	72 824 (74.9)	40 358 (68.6)
Yes	14 706 (35.4)	16 599 (30.8)	16 139 (29.1)	24 351 (25.1)	18 445 (31.4)
Psychiatric medication in toxicology testing^b^					
Antidepressants					
Present	17 551 (50.5)	3288 (10.3)	819 (2.4)	824 (2.3)	8642 (21.1)
Not present	9939 (28.6)	9821 (30.7)	14 123 (41.3)	12 526 (35.3)	18 022 (44)
Not tested	7259 (20.9)	18 885 (59.0)	19 235 (56.3)	22 181 (62.4)	14 327 (35)
Anticonvulsants					
Present	4366 (19.1)	129 (1.0)	120 (0.8)	132 (0.9)	3000 (9.8)
Not present	10 626 (46.4)	4254 (33.1)	5825 (38.2)	5190 (36.7)	13 802 (45)
Not tested	7893 (34.5)	8477 (65.9)	9303 (61.0)	8802 (62.3)	13 839 (45.2)
Antipsychotics					
Present	3693 (16.2)	114 (0.9)	38 (0.2)	39 (0.3)	1406 (4.7)
Not present	11 367 (49.7)	4480 (34.6)	6101 (39.9)	5243 (37)	14 787 (49.1)
Not tested	7801 (34.1)	8347 (64.5)	9142 (59.8)	8894 (62.7)	13 937 (46.3)
Benzodiazepines					
Present	10 930 (41.5)	729 (5.3)	769 (4.7)	534 (3.5)	10 134 (27.9)
Not present	13 535 (51.4)	7177 (52.4)	10 436 (63.4)	9334 (61.5)	22 143 (60.9)
Not tested	1889 (7.2)	5778 (42.2)	5245 (31.9)	5300 (34.9)	4066 (11.2)

^a^ No indicates no/not available/unknown as originally provided by the National Violent Death Record System.

^b^ Data were combined for whether the specific substance was tested (tested, not tested, unknown) and the results of the toxicology tests. Percentages were calculated with all suicide decedents. Column totals do not sum to 100% owing to missing data (coded as unknown for whether the substance was tested).

**Table 3.** Multivariable Logistic Regression Models With Suicide Intent Disclosure and Suicide Note Leaving as Outcomes

	Odds ratio (95% CI)	
	No recent suicide intent disclosure	No suicide note
Class		
1: Mental health and substance problems	1 [Reference]	1 [Reference]
2: Mental health problems	1.04 (1.01-1.07)	1.15 (1.12-1.18)
3: Crisis, alcohol-related, and intimate partner problems	1.16 (1.12-1.19)	1.10 (1.07-1.13)
4: Physical health problems	2.58 (2.51-2.66)	1.45 (1.41-1.49)
5: Polysubstance problems	1.62 (1.57-1.66)	1.09 (1.06-1.12)
Age, y		
≤10	1 [Reference]	1 [Reference]
11-15	1.22 (0.82-1.80)	0.32 (0.19-0.54)
16-24	0.98 (0.66-1.44)	0.48 (0.29-0.80)
25-40	1.03 (0.70-1.52)	0.61 (0.37-1.01)
41-54	1.22 (0.83-1.79)	0.50 (0.30-0.83)
55-70	1.39 (0.94-2.05)	0.45 (0.27-0.75)
≥71	1.04 (0.71-1.53)	0.46 (0.28-0.76)
Sex		
Male	1 [Reference]	1 [Reference]
Female	1.08 (1.06-1.10)	0.72 (0.71-0.74)
Education level		
High school or above	1 [Reference]	1 [Reference]
Below high school	0.87 (0.84-0.89)	1.50 (1.45-1.55)
Race and ethnicity		
American Indian or Alaska Native^a^	0.86 (0.80-0.93)	1.70 (1.56-1.85)
Asian or Other Pacific Islander^a^	1.31 (1.23-1.40)	1.05 (1.0003-1.11)
Black or African American^a^	1.27 (1.22-1.32)	1.88 (1.81-1.95)
Hispanic	1.02 (0.99-1.06)	1.39 (1.34-1.44)
Other/unspecified^a^	1.88 (1.53-2.30)	1.70 (1.44-2.01)
≥2 Races^a^	0.83 (0.77-0.90)	0.99 (0.92-1.06)
White^a^	1 [Reference]	1 [Reference]
Marital status		
Married, civil union, domestic partnership	1 [Reference]	1 [Reference]
Divorced	0.98 (0.96-1.003)	0.81 (0.79-0.83)
Married, civil union, domestic partnership but separated	0.80 (0.76-0.84)	0.63 (0.60-0.66)
Never married	1.22 (1.19-1.25)	0.85 (0.83-0.87)
Single, not otherwise specified	1.17 (1.09-1.27)	0.90 (0.84-0.97)
Widowed	1.03 (0.99-1.08)	0.83 (0.80-0.86)
Military affiliation		
Yes	0.96 (0.93-0.98)	0.93 (0.91-0.95)
No	1 [Reference]	1 [Reference]
Residence		
Nonmetropolitan	1.01 (0.99-1.03)	1.28 (1.25-1.30)
Metropolitan	1 [Reference]	1 [Reference]

^a^ Non-Hispanic ethnicity.

### Demographic Disparities

Compared with White individuals, Black individuals had higher odds of nondisclosure of intent (OR, 1.27; 95% CI, 1.22-1.32) and no note (OR, 1.88; 95% CI, 1.81-1.95). Females were less likely to disclose intent (OR, 1.08; 95% CI, 1.06-1.10) and more likely to leave a note (OR, 0.72; 95% CI, 0.71-0.74).

## Discussion

We identified 5 suicide profiles, each requiring tailored prevention strategies. Class 1 had comorbid mental and substance use disorders. Class 2 was dominated by mental disorders. Class 3 experienced crisis, alcohol-related, and intimate partner problems; class 4 displayed physical health problems; and class 5 displayed substance-use problems. We found class 4 was least likely to disclose suicide intent, leave suicide notes as intent indicators, and have known mental illness or psychotropics detected by toxicology.

Class 4 emerged as the most concerning group: the largest (31.7%) and increasing the most (31.2% increase since 2016). With the strikingly lowest rate of diagnosed mental disorders and toxicology-detected psychotropic medications, class 4 comes the closest to having an “invisible suicide decedent risk profile.” Several factors may explain this. Men’s suicide rate is 4 times higher than women’s, and men are poorer at help-seeking. Physical illnesses can cause physical pain and psychological distress, which can lead to feelings of helplessness and heighten suicide risk that may be overlooked in medical settings.^42^ Suicidal patients may prioritize physical over mental health care because they consulted non–mental health care professionals twice as often as mental health professionals in the 30 days before death by suicide. Therefore, nonpsychiatric health care settings, especially primary care and emergency departments, are crucial for suicide risk screening.^43^

The composition of class 4 (predominantly older adults, non-Hispanic Asian American individuals, and veterans) reflects broader suicide trends in these populations.^44^ Systemic barriers such as stigma and social isolation may impede the recognition and treatment of mental health issues.^10^ Suicide decedents in class 4 were more likely to die on the first attempt because they mostly used firearms.^5^ The contrast in psychotropic medication usage between class 4 and class 1 is noteworthy. In class 4, only 2.3% of individuals were using antidepressants, compared with 50.5% in class 1. This discrepancy raises concerns about the potential undertreatment of major depression, given its link to nearly half of US suicide deaths.^5^ This highlights the urgent need to enhance the diagnosis and treatment of major depression in health care settings.^5^ Future research is needed to understand why class 4 has grown as a proportion of suicide decedents over time because that may identify other suicide prevention targets.

In contrast, while both classes 1 and 2 experienced high rates of mental health problems, they exhibited distinct challenges. Class 1 faced the additional challenge of comorbid substance use. A significant majority of class 1 members (84.8%-99.7%) had received or were receiving mental illness treatment.^45^ Notably, treatment rates escalated between 2011 and 2015, paralleling the increase in opioid-involved overdose deaths, which surged from 22 784 in 2011 to 68 630 in 2020.^46^ The increase in prescription and illegal opioids, particularly fentanyl, and its association with poisoning-related suicides in class 1 necessitates further investigation. Toxicology findings indicate that nearly half of the suicide decedents in class 1 were not taking psychotropic medications at the time of death, indicating possible nonadherence or cessation of treatment before suicide.^47^ In contrast, class 2, with minimal substance use disorders and less mental health treatment than class 1, experienced a significant decrease in suicide rates, suggesting improvements in mental health care, but limited resources for managing substance use disorders were underlying the trends in these 2 classes of suicide.

Class 5, marked by widespread polysubstance problems, grew the fastest. Toxicology reports revealed one-fifth of class 5 decedents had no current psychotropic medication at the time of death, and only 3% had a treatment history. This class also recorded the highest rates of positive test results for substances such as amphetamine (12.5%), alcohol (39.6%), marijuana (19.5%), and cocaine (7.9%). Challenges like stigma and health complications from multiple substance dependencies likely hinder recovery efforts, leading to lower treatment engagement and adherence and a hesitancy to reveal suicidal tendencies.^48^ As indicated by the trends in classes 1 and 2, systemic barriers^49^ to accessing substance abuse treatment, and potentially the absence of culturally tailored and gender-specific programs, may have contributed to the suicide risk in classes 1 and 5.

Following prior studies, we found class 3 represented younger males struggling with alcohol misuse amid economic challenges^50^ and separated or single males facing intimate partner relationship difficulties.^51^ Prevention should extend beyond immediate, visible aspects of these crises to comprehensively address less apparent but underlying causes often overlooked in research and intervention strategies.

### Demographic Differences

The demographic characteristics of class 4, comprising an aging, rural, racial and ethnic minority, widowed, veteran, and less educated population, suggest that shared factors (eg, limited access to education/health care, social isolation, financial difficulties, trauma, low health literacy) may have contributed to neglected suicide risks and increase physical-illness–related suicide rates observed from 2016 to 2020.^6,44^ The post-2011 increase of class 5 may be partially explained by societal changes affecting this high school–educated, single/never-married, 25- to 54-year-old male demographic segment, including economic stress from uneven recovery after the Great Recession, increased substance availability,^52^ and absence of protective marriage.^53^ A higher proportion of divorced individuals (24.7%) and females (42.3%) in class 1 suggests that relationship breakdowns are significant stressors, as divorce is often associated with psychological and substance use problems.^54^ The observed greater reluctance among females to disclose suicidal intent was unexpected, presenting an area for further investigation. Class 3, with a notable majority of males (88.5%), aligns with previous findings linking males to heightened crisis, alcohol-related issues, and intimate partner problems.^3,5,17^

### Research and Policy Implications to Improve Suicide Prevention

Our findings reinforce the need for tailored approaches that recognize the diverse needs of the suicide decedent profile. The 5 suicide profiles based on postmortem data can also inform clinicians to screen for each suicide risk subtype for personalized suicide prevention strategies. For class 4, integrated care coordinating physical and mental health services is crucial, especially in primary care/internal medicine settings,^55^ where a significant proportion of patients experience at least 1 (60%) or 2 (40%) chronic diseases,^56^ including diabetes (37.3 million people), Alzheimer disease (5.8 million people), and cancer (1.6 million people).

Class 1 is characterized by significant comorbid mental health and substance use problems, calling for a holistic approach that integrates psychiatric and substance abuse treatment,^57^ medication management,^58^ and community-based rehabilitation to foster recovery and social reintegration.^59^ Class 2 might benefit from comprehensive mental health services,^60^ including psychoeducation tailored to individuals and families.^61^ Improved medication adherence is potentially helpful but not the only option. Beyond medication, therapies such as cognitive-behavioral therapy^62^ and dialectical behavior therapy^63^ can be effective for suicidal behaviors, supporting risk reduction and enhanced coping mechanisms, particularly for those with depression and borderline personality disorder.^62^

For class 3, interventions like safety planning, crisis intervention,^64^ alcohol treatment programs,^65^ and family/relationship counseling are imperative.^66^ Class 5, characterized by polysubstance issues, could benefit from mental health screenings, substance use treatment,^52^ harm reduction,^67^ and peer support.^68^ Both classes 3 and 5 may also gain from treatments addressing the overlap of suicide risks and substance use, as well as firearm control measures.

Given the increasing diversity in the US, clinical interventions should be culturally relevant and sensitive, accommodating different racial and ethnic, sex, age, and educational backgrounds. Such inclusivity ensures that interventions are effective and resonate with diverse groups.^6,22,26,69,70^

### Strengths and Limitations

This study harnessed comprehensive suicide data to identify 5 suicide profiles, each informing unique prevention strategies. This study has limitations. First, the NVDRS data revealed geographic variations due to decentralized coroner systems and diverse law enforcement practices. Combined with variable experience levels among data abstractors and reliance on second-hand information, these variations may introduce data incompleteness. To mitigate this, we applied fixed effects and county-year interactions to control for location and time-specific variations. Our conclusions remained robust.

Second, data coding, particularly in ambiguous categories like no/not available/unknown, may affect the accuracy of our findings. This is particularly true for toxicology data, where underreporting is common. We tackled this by examining drug testing details and cross-validating our findings through multiple imputations and direct data comparison.

Third, the potential for misclassifying suicide deaths and underreporting previous suicide attempts poses a risk of overestimating the incidence of first-attempt suicides. This challenge is amplified by reliance on medical examiner and law enforcement reports, which may not always capture the full history of attempts.^1^ Our high rate of first-attempt decedents (60%-94%) aligns with other NVDRS studies (79%)^33^ but contrasts with lower rates reported in some psychological autopsy studies (10%-40%).^8,10,71,72^

Fourth, the high frequency of firearm-related deaths in the US limits the generalizability of our findings to countries with lower firearm availability.^5,6,73^ With the highest per capita gun ownership (45%) in the developed world,^74^ the US presents a unique context for firearm suicide by making firearms accessible to many at-risk individuals.

Finally, there is a risk of biased screening related to demographics.^75^ For example, Black and Hispanic individuals may receive less comprehensive investigations than White individuals, affecting data reliability.^75^ Given possible unreliable data, we suggest interpreting our findings as part of a broader approach to guide suicide prevention.

Despite these limitations, the NVDRS remains the only national US dataset that comprehensively captures circumstances, toxicology, and suicide methods.^13^ We found internally consistent results, despite dataset weaknesses, offering a nuanced understanding of suicide.

## Conclusions

This study identified 5 distinct suicide profiles, underscoring the complexity of suicide and illustrating its multifaceted nature. A notable finding is the rise of an invisible risk profile characterized by physical health problems, highlighting psychiatric underdiagnosis and subsequent undertreatment. These findings highlight the need for tailored suicide prevention strategies that address the specific needs of each profile to the extent they are identifiable, or prevent impulsive thoughts from being acted on, moving away from a 1-size-fits-all approach. Improving the detection and treatment of coexisting mental health conditions, substance and alcohol use disorders, and physical illnesses is paramount. Moreover, the implementation of means restriction strategies plays a vital role in reducing suicide risks across most of the profiles, reinforcing the need for a multifaceted approach to suicide prevention.

## Notes

Supplement 1. **eMethods 1.** National Violent Death Reporting System (NVDRS) Data: Unique Value and Fit for the Current Study **eMethods 2.** Rationale Behind the Selection of Indicators in the National Violent Death Reporting System (NVDRS) to Identify Suicide Death Profiles using Latent Class Analysis **eMethods 3.** Reliability and Possible Misclassification Scenarios in the National Violent Death Reporting System (NVDRS) Data **eMethods 4.** Strategies to Deal with Missing Data and Misclassification Error **eMethods 5.** Addressing the Implications of Unbalanced Panel Data on Study Outcomes in the National Violent Death Reporting System (NVDRS) Data **eFigure 1.** Scree Plot of Bayesian Information Criterion (BIC) Values Across Ten Latent Class Analysis Solutions **eFigure 2.** Two-Class Latent Class Solution **eFigure 3.** Three-Class Latent Class Solution **eFigure 4.** Four-Class Latent Class Solution **eFigure 5.** Five-Class Latent Class Solution **eFigure 6.** Six-Class Latent Class Solution **eFigure 7.** Seven-Class Latent Class Solution **eFigure 8.** Eight-Class Latent Class Solution **eFigure 9.** Nine-Class Latent Class Solution **eFigure 10.** Ten-Class Latent Class Solution **eTable 1.** Indicator Information including Definition, Original Response Categories, and Recoding Rules **eTable 2.** Model Fit Statistics of Latent Class Analysis Models **eTable 3.** Distribution of Precipitating Circumstances by Five Suicide Decedent Profiles in the U.S., 2003-2020 (N=306 800) **eTable 4.** Results of Analysis without Missing Data Imputation **eTable 5.** Class Prevalence by Year **eTable 6.** Results of Analysis without Missing Data Imputation Additionally controlling for Fixed Effects of Year and State
eReferences

Supplement 2. Data sharing statement
