# Supplementary material for: Decoding Suicide Decedent Profiles and Signs of Suicidal Intent Using Latent Class Analysis
Source: JAMA Psychiatry. 2024 Mar 20;81(6):595–605. doi: 10.1001/jamapsychiatry.2024.0171 (PMC10955339; doi:10.1001/jamapsychiatry.2024.0171)
Supplement: Supplement 1. — eMethods 1. National Violent Death Reporting System (NVDRS) Data: Unique Value and Fit for the Current Study eMethods 2. Rationale Behind the Selection of Indicators in the National Violent Death Reporting System (NVDRS) to Identify Suicide Death Profiles using Latent Class Analysis eMethods 3. Reliability and Possible Misclassification Scenarios in the National Violent Death Reporting System (NVDRS) Data eMethods 4. Strategies to Deal with Missing Data and Misclassification Error eMethods 5. Addressing the Implications of Unbalanced Panel Data on Study Outcomes in the National Violent Death Reporting System (NVDRS) Data eFigure 1. Scree Plot of Bayesian Information Criterion (BIC) Values Across Ten Latent Class Analysis Solutions eFigure 2. Two-Class Latent Class Solution eFigure 3. Three-Class Latent Class Solution eFigure 4. Four-Class Latent Class Solution eFigure 5. Five-Class Latent Class Solution eFigure 6. Six-Class Latent Class Solution eFigure 7. Seven-Class Latent Class Solution eFigure 8. Eight-Class Latent Class Solution eFigure 9. Nine-Class Latent Class Solution eFigure 10. Ten-Class Latent Class Solution eTable 1. Indicator Information including Definition, Original Response Categories, and Recoding Rules eTable 2. Model Fit Statistics of Latent Class Analysis Models eTable 3. Distribution of Precipitating Circumstances by Five Suicide Decedent Profiles in the U.S., 2003-2020 (N=306 800) eTable 4. Results of Analysis without Missing Data Imputation eTable 5. Class Prevalence by Year eTable 6. Results of Analysis without Missing Data Imputation Additionally controlling for Fixed Effects of Year and State eReferences [file jamapsychiatry-e240171-s001.pdf]

Supplemental Online Content

Xiao Y, Bi K, Yip PSF, et al. Decoding suicide decedent profiles and signs of suicidal intent using latent class analysis. *JAMA Psychiatry.* Published online March 20, 2024. doi:10.1001/jamapsychiatry.2024.0171

eMethods 1. National Violent Death Reporting System (NVDRS) Data: Unique Value and Fit for the Current Study
eMethods 2. Rationale Behind the Selection of Indicators in the National Violent Death Reporting System (NVDRS) to Identify Suicide Death Profiles using Latent Class Analysis
eMethods 3. Reliability and Possible Misclassification Scenarios in the National Violent Death Reporting System (NVDRS) Data
eMethods 4. Strategies to Deal with Missing Data and Misclassification Error
eMethods 5. Addressing the Implications of Unbalanced Panel Data on Study Outcomes in the National Violent Death Reporting System (NVDRS) Data
eFigure 1. Scree Plot of Bayesian Information Criterion (BIC) Values Across Ten Latent Class Analysis Solutions
eFigure 2. Two-Class Latent Class Solution
eFigure 3. Three-Class Latent Class Solution
eFigure 4. Four-Class Latent Class Solution
eFigure 5. Five-Class Latent Class Solution
eFigure 6. Six-Class Latent Class Solution
eFigure 7. Seven-Class Latent Class Solution
eFigure 8. Eight-Class Latent Class Solution
eFigure 9. Nine-Class Latent Class Solution
eFigure 10. Ten-Class Latent Class Solution
eTable 1. Indicator Information including Definition, Original Response Categories, and Recoding Rules
eTable 2. Model Fit Statistics of Latent Class Analysis Models
eTable 3. Distribution of Precipitating Circumstances by Five Suicide Decedent Profiles in the U.S., 2003-2020 (N=306 800)
eTable 4. Results of Analysis without Missing Data Imputation
eTable 5. Class Prevalence by Year
eTable 6. Results of Analysis without Missing Data Imputation Additionally controlling for Fixed Effects of Year and State
eReferences

This supplemental material has been provided by the authors to give readers additional information about their work.

© 2024 Xiao Y et al. *JAMA Psychiatry.*

eMethods 1. National Violent Death Reporting System (NVDRS) Data: Unique Value and Fit for the Current Study

NVDRS is uniquely suitable to answer the research question of this study, which is to identify distinct suicide death profiles.

1. NVDRS is the first system to accumulate and integrate comprehensive investigative data from multiple sources, encompassing law enforcement agencies, forensic medical examiners, toxicological analyses, and death certification records. Importantly, the NVDRS dataset encapsulates the three pivotal domains of interest for this study: circumstances, toxicology, and methods. Through this integrated dataset, the NVDRS offers holistic and detailed information on circumstances precipitating suicide deaths.
2. NVDRS operates on a national scale. NVDRS began in 2003 with six participating states (Maryland, Massachusetts, New Jersey, Oregon, South Carolina, and Virginia) and has expanded incrementally over time to all 50 states, the District of Columbia, and Puerto Rico in 2018.
3. NVDRS data are updated annually.
4. NVDRS has relatively complete vital data due to the partnerships among state VDRS programs and local health departments. As part of an active surveillance system, VDRS programs work closely with local vital registrars' offices to identify deaths that meet the NVDRS case definition and to avoid cases being missed or inappropriately included. The CDC also monitors case ascertainment and variable completeness through regular technical assistance calls, which include reviews of the internal data quality dashboard in the web-based system that are updated in real-time. Overall, core variables that represent demographic characteristics (e.g., age, sex, race, and ethnicity) and manner of death were known for >99% of cases.

© 2024 Xiao Y et al. *JAMA Psychiatry.*

eMethods 2. Rationale Behind the Selection of Indicators in the National Violent Death Reporting System (NVDRS) to Identify Suicide Death Profiles using Latent Class Analysis

Principles and the Importance of Selecting Indicators for Latent Class Analysis (LCA)
Selecting the indicators in the LCA is critical for identifying theory-guided and empirical evidence-guided comprehensive suicide death profiles, as well as providing a basis for future application in other contexts. To this end, we selected three main domains of indicators: (1) 19 circumstances/precipitating factors of suicide, (2) 6 toxicology variables, and (3) 1 weapon (or suicide method) variable.

Circumstances
Information about circumstances is important to help inform prevention activities. This information is gathered from witnesses, family, friends, and other informants by death investigators.[1]

We screened all circumstance indicators and selected indicators that have been consistently measured throughout the whole study observation period (from 2003 to 2020). Indicators added to the NVDRS after 2003 were excluded, such as TreatmentNonAdherence_c (added in 2020), AbusedAsChild_c (added in 2009), despite their importance to understanding suicide risks.

The variable that recorded the suicide decedent's family relationship problems (familyrelationship_c) was added in August 2013.[1] Previously, family relationship problems were coded under the variable "relationshipproblemother_c," which recorded any relationship problems other than relationship problems with intimate partners. As a result, we combined the family relationship problems (familyrelationship_c) and other relationship problems (relationshipproblemother_c) to create a new relationship problem variable ("other relationship problem"), which captured all relationship problems (other than intimate partners) throughout the study observation period (2003-2020).

Toxicology
Toxicology information is important to collect in order to understand the role of alcohol, illicit drugs, and prescription drugs in violent deaths.[1]

Weapon (Suicide methods)
Weapon in NVDRS refers to the objects (e.g., instruments like guns) used to injure and actions (e.g., behaviors like setting fires) that lead to injury.[1]

For poisonings, NVDRS tracks information on individual poisonous substances in the toxicology section, including the poison that killed the victim. Poisoning (e.g., fatal ingestion or injection of an illicit drug, alcohol, pharmaceutical, carbon monoxide, gas, rat poison, or insecticide) was defined by the latest MMWR (2020).[2]

We provide detailed illustrations for the data considerations (eMethods 2 and 3) and analytic considerations (eMethods 4 and 5).

© 2024 Xiao Y et al. *JAMA Psychiatry.*

eMethods 3. Reliability and Possible Misclassification Scenarios in the National Violent Death Reporting System (NVDRS) Data

The National Violent Death Reporting System (NVDRS) is an essential source of comprehensive information about violent deaths, facilitating the understanding of patterns, circumstances, and causes surrounding such occurrences. Despite its importance, like many other national surveillance databases, NVDRS has limitations related to data reliability and potential misclassification.

Reliability
The NVDRS incorporates a diverse set of data from multiple sources, including law enforcement, coroner/medical examiners, and toxicology. To maintain consistency, NVDRS employs rigorous coding mechanisms designed to improve its reliability, standardize varied data inputs, and enhance ensuring clarity, accuracy, and usefulness throughout the dataset.

Misclassification Scenarios:
Given the diverse data sources, potential misclassification remains a pertinent concern. For instance, law enforcement reports may not always align with medical examinations, leading to discrepancies in the cause or nature of the violent death. NVDRS may also contain subjective information, especially concerning mental health, and can be prone to biases or inaccuracies based on the personal perspectives of those reporting.

Other threats to the reliability of NVDRS data include:

1. Limited Geographic Representativeness: NVDRS data, though currently including all 50 states, does omit some counties. For example, some large states like California and Texas offer data only from select counties.*
2. Dependence on Collaborative Partnerships: The quality and timeliness of data rely heavily on collaborations between the VDRS programs and various stakeholders, including local health departments and law enforcement. Some regions, especially those with decentralized coroner systems or multiple law enforcement jurisdictions, might face challenges in fostering seamless data-sharing relations.
3. Inconsistencies in Toxicology Data: Toxicology data collection varies across regions and categories, influencing the reliability of substance detection metrics.
4. Data Source Limitations: Data abstractors can only utilize available investigative reports, which might sometimes be incomplete or delayed, especially in cases of legal interventions or homicides. The precipitating circumstance of mental health treatment history is dependent upon access to mental health treatment.
5. Varied Case Definitions: Discrepancies can arise when different sources categorize a death differently. NVDRS abstractors aim to harmonize these differences using standardized definitions.
6. Variations in Data Coding: Coding discrepancies can emerge based on the expertise of the abstractor. To mitigate this, CDC offers guidance, training, and a coding manual to promote uniform data collection.
7. Second-hand Medical Data: Information related to medical and mental health often originates from secondary sources, like family members or coroner records, rather than direct medical records. This can impact the accuracy and completeness of such data.

* Of the participating VDRS programs, 46 states (Alabama, Alaska, Arizona, Arkansas, Colorado, Connecticut, Delaware, Georgia, Idaho, Illinois, Indiana, Iowa, Kansas, Kentucky, Louisiana, Maine, Maryland, Massachusetts, Michigan, Minnesota, Mississippi, Missouri, Montana, Nebraska, Nevada, New Hampshire, New Jersey, New Mexico, New York, North Carolina, North Dakota, Ohio, Oklahoma, Oregon, Pennsylvania, Rhode Island, South Carolina, South Dakota, Tennessee, Utah, Vermont, Virginia, Washington, West Virginia, Wisconsin, and Wyoming) collected information on all violent deaths that occurred in their state in 2020. In addition, data were collected on all violent deaths that occurred in the District of Columbia and Puerto Rico in 2020. Two states, California and Texas, joined NVDRS with plans to collect data on violent deaths in a subset of counties. While California collected data from death certificates for all violent deaths in the state in 2020 (n = 6,902), data for violent deaths that occurred in 35 counties (Amador, Butte, Colusa, Contra Costa, Fresno, Glenn, Humboldt, Imperial, Kern, Kings, Lassen, Lake, Los Angeles, Marin, Mendocino, Merced, Modoc, Mono, Orange, Placer, Sacramento, San Benito, San Diego, San Francisco, San Mateo, Santa Cruz, Shasta, Siskiyou, Solano, Sonoma, Stanislaus,

© 2024 Xiao Y et al. *JAMA Psychiatry.*

Tehama, Trinity, Ventura, and Yolo) also included information from coroner or medical examiner records and law enforcement reports and are included throughout the rest of the report (n = 4,672; 67.7%). These 35 counties represented 71% of California's population (9). While Texas also collected data from death certificates for all violent deaths in the state in 2020 (n = 6,564), data for violent deaths that occurred in four counties (Bexar, Dallas, Harris, and Tarrant) also included information from coroner or medical examiner records and law enforcement reports and are included throughout the rest of the report (n = 2,737; 41.7%). These four counties represented 39% of the state's population (9). Because <100% of violent deaths were abstracted, data from California and Texas used in this study do not represent all violent deaths occurring in these states.

© 2024 Xiao Y et al. *JAMA Psychiatry.*

eMethods 4. Strategies to Deal with Missing Data and Misclassification Error

Random Misclassification Error Due to Negative Ambiguity

With regard to the 19 circumstance variables, the source data from the CDC combined the category "No" with "Not Available" and "Unknown". We had no ability to separate these categories. We thus had complete data for all suicide cases with two levels (1 = Yes/Present 2 = No/Not present/Unknown).

For the 5 specific toxicology variables, the original coding of these variables was of four levels: 1 = Present; 2 = Not present; 8 = Not Applicable; 9 = Unknown, along with relatively large amounts of empty cells (i.e., missing values). To make these five variables' coding consistent with the 19 circumstance variables, we followed the procedure of Culbreth et al. (2021) and did the following recoding: (a) recoded 2 (Not present) as 0; (b) recoded 8 (Not applicable) and 9 (Unknown) as 0; and (c) recoded empty cells as 0. For the numeric number of substance variable, we treated it as a categorical variable and then collapsed 4 or more numbers of substances into one level, and collapsed empty cells and 0 into one level, resulting in a total of five levels: Level 1 = 0/Missing, Level 2 = 1, Level 3 = 2, Level 4 = 3, Level 5 = 4 or 4+.

For the weapon variable (suicide method), we grouped the less prevalent suicide methods into a level named "Other". We collapsed empty cells (missing values) and the "Other" level into one level named "Other/Unknown".

Statistical Impact of Misclassification Error

A response of "No" typically indicates that the particular outcome or characteristic was screened for and found to be absent, thus representing a true negative result. However, "Not Available" and "Unknown" are not synonymous with a true negative. "Not Available" can imply that the screening or testing was not performed, or that the data was not recorded or is missing for other reasons. "Unknown" could mean that the information is undetermined, inconclusive, or uncertain. We suggest this ambiguity is random and that, in general, reasonable good-faith efforts have been made to collect the relevant information such that the lack of a definitive "No" was not due to policy, but rather, to county-level (e.g., staffing of medical/legal examiners, training, coding habits) and temporal (e.g., NVDRS renewed yearly with lags of suicide death reports) variations..

Thus, our logistic regression models have misclassification errors in the dependent variable (non-disclosure of suicidal intent/no suicide note) and in the independent variables (the 5 classes derived from LCA). We address misclassification in the dependent variable and independent variables separately.

Misclassification error in the dependent variable of logistic regression is similar to a specific form of omitted variable bias.[1] To the extent the omitted variables regarding entries of "Not Available" and "Unknown" in the dependent variable are due to patterns of decisions made by county-level law enforcement and/or medical personnel regarding the collection and entering of these data, we included county-fixed effects, year fixed effects, and interactions of the county indicators and year indicators. This will largely correct for bias due to county-level variation in data collection and personnel changes over time. In other words, this picks up any unintended deviations from the NVDRS data collection standards that personnel are trained to follow.
With respect to random measurement error in the independent variables, the impact will be to attenuate the estimated coefficients.[2] In other words, our results were conservative (smaller than the case where random measurement error was not present).

Imputation of Missing Data

For missing values that were not combined with true negative values, we used Little's Test to examine the missing patterns through the Naniar package in R. Results showed missing at random. However, we also re-ran our analysis without multiple imputations. Results, as shown in eTable 3, were validated and consistent with the main statistics we presented in the main text. In addition, we controlled for the fixed effect of state and incident year using the same non-imputed data, and the results were broadly consistent (eTable 5).

© 2024 Xiao Y et al. *JAMA Psychiatry.*

Future studies could benefit from explicit categorization and separate analysis of these response types, to provide a more accurate picture of the distribution of the outcomes or characteristics under study. Furthermore, statistical techniques such as multiple imputation can be used to handle missing data, which could help to reduce the bias introduced by missing or inconclusive responses.

© 2024 Xiao Y et al. *JAMA Psychiatry.*

eMethods 5. Addressing the Implications of Unbalanced Panel Data on Study Outcomes in the National Violent Death Reporting System (NVDRS) Data

States joined the NVDRS at different years (see below)[3], making our data unbalanced panel data. However, such data format will not affect the study outcomes, because we focus on individual-level potential risks precipitating suicide deaths, and the indicators we selected to characterize the suicide death profiles have been consistently included in the NVDRS data from 2003 to 2020.

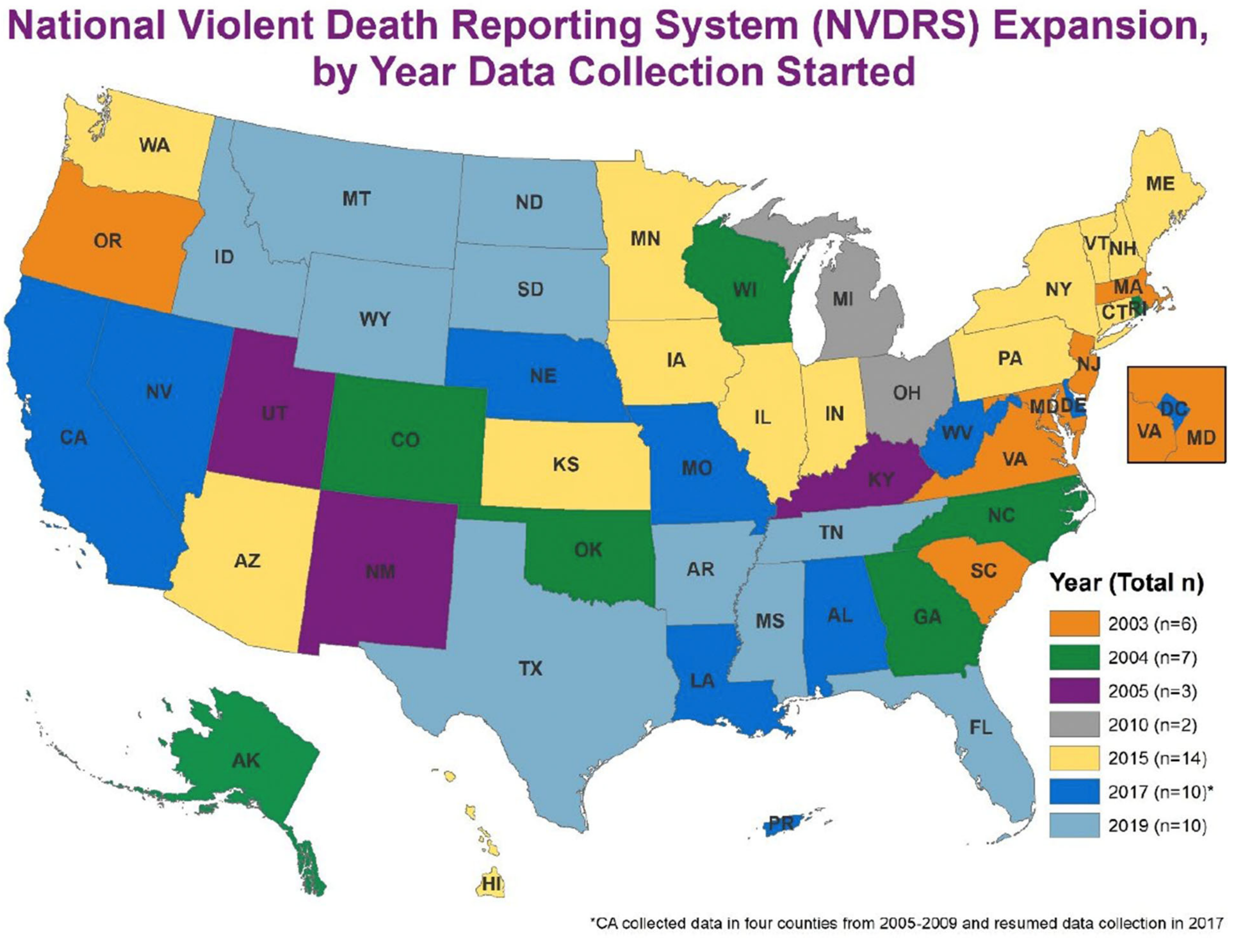

NVDRS User Guidelines

VDRS Program	2003	2004	2005	2006	2007	2008	2009	2010	2011	2012	2013	2014	2015	2016	2017	2018	2019	2020
Alabama																X	X	X
Alaska	X	X	X	X	X	X	X	X	X	X	X	X	X	X	X	X	X	X
Arizona													X	X	X	X	X	X
Arkansas																		X
California															X[a]	X[b]	X[c]	X[d]
Colorado		X	X	X	X	X	X	X	X	X	X	X	X	X	X	X	X	X
Connecticut													X	X	X	X	X	X
Delaware															X	X	X	X
District of Columbia															X	X	X	X

© 2024 Xiao Y et al. *JAMA Psychiatry.*

**Florida**																		
**Georgia**		X	X	X	X	X	X	X	X	X	X	X	X	X	X	X	X	X
**Hawaii**													X	X	[e]	[e]	X	[e]
**Idaho**																		X
**Illinois**														X[f]	X[f]	X[f]	X[f]	X
**Indiana**														X	X	X	X	X
**Iowa**														X	X	X	X	X
**Kansas**													X	X	X	X	X	X
**Kentucky**			X	X	X	X	X	X	X	X	X	X	X	X	X	X	X	X
**Louisiana**																X	X	X
**Maine**													X	X	X	X	X	X
**Maryland**	X	X	X	X	X	X	X	X	X	X	X	X	X	X	X	X	X	X
**Massachusetts**	X	X	X	X	X	X	X	X	X	X	X	X	X	X	X	X	X	X
**Michigan**												X	X	X	X	X	X	X
**Minnesota**													X	X	X	X	X	X
**Mississippi**																		X
**Missouri**																X	X	X
**Montana**																	X	X
**Nebraska**																X	X	X
**Nevada**															X	X	X	X
**New Hampshire**													X	X	X	X	X	X
**New Jersey**	X	X	X	X	X	X	X	X	X	X	X	X	X	X	X	X	X	X
**New Mexico**			X	X	X	X	X	X	X	X	X	X	X	X	X	X	X	X
**New York**													X	X	X	X	[g]	X
**North Carolina**		X	X	X	X	X	X	X	X	X	X	X	X	X	X	X	X	X
**North Dakota**																	X	X
**Ohio**									X	X	X	X	X	X	X	X	X	X
**Oklahoma**		X	X	X	X	X	X	X	X	X	X	X	X	X	X	X	X	X
**Oregon**	X	X	X	X	X	X	X	X	X	X	X	X	X	X	X	X	X	X
**Pennsylvania**														X[f]	X[f]	X[f]	X[f]	X
**Puerto Rico**															X	X	X	X
**Rhode Island**		X	X	X	X	X	X	X	X	X	X	X	X	X	X	X	X	X
**South Carolina**	X	X	X	X	X	X	X	X	X	X	X	X	X	X	X	X	X	X
**South Dakota**																		X
**Tennessee**																		X
**Texas**																		X[h]
**Utah**			X	X	X	X	X	X	X	X	X	X	X	X	X	X	X	X
**Vermont**													X	X	X	X	X	X
**Virginia**	X	X	X	X	X	X	X	X	X	X	X	X	X	X	X	X	X	X

© 2024 Xiao Y et al. *JAMA Psychiatry.*

**Washington**														X[f]	X[f]	X	X	X
**West Virginia**															X	X	X	X
**Wisconsin**		X	X	X	X	X	X	X	X	X	X	X	X	X	X	X	X	X
**Wyoming**																	X	X
**TOTAL**	**7**	**13**	**16**	**16**	**16**	**16**	**16**	**16**	**17**	**17**	**17**	**18**	**27**	**32**	**37**	**41**	**44**	**50**

© 2024 Xiao Y et al. *JAMA Psychiatry.*

eFigure 1. Scree Plot of Bayesian Information Criterion (BIC) Values Across Ten Latent Class Analysis Solutions

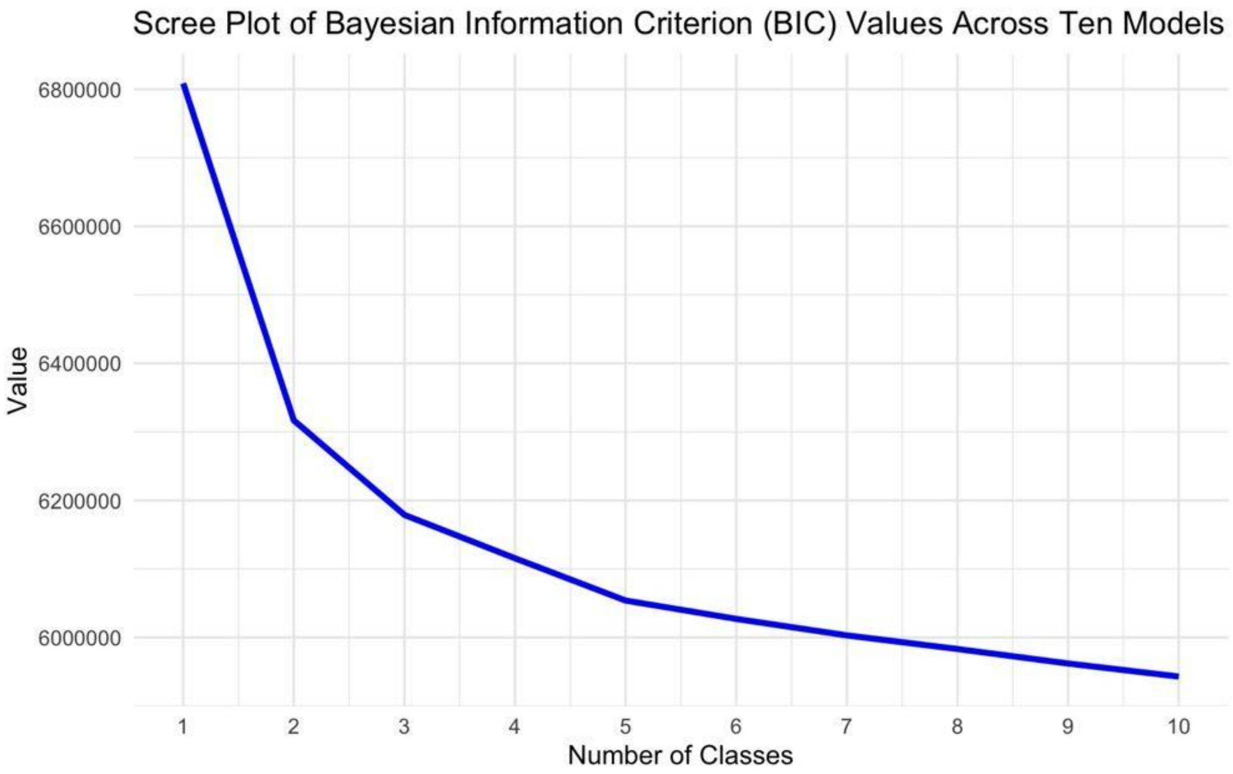

© 2024 Xiao Y et al. *JAMA Psychiatry.*

eFigure 2. Two-Class Latent Class Solution

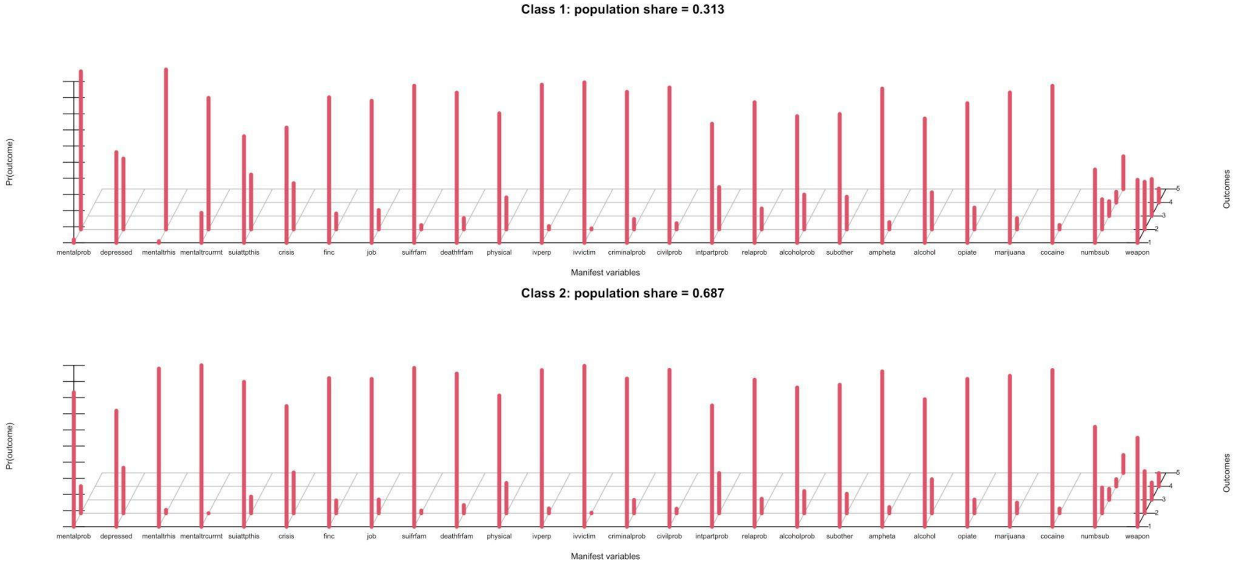

Note. mentalprob = Mental Health Problem; depressed = Depressed Mood; mentaltrhis = History Mental Illness Treatment; mentaltrcurrnt = Mental Illness Treatment Current; suiattpthis = Suicide Attempt History; crisis = Any Crisis; finc = Financial Problem; job = Job Problem; suifrfam = Recent Suicide Friend Family; deathfrfam = Death Friend Or Family Other; physical = Physical Health Problem; ivperp = Interpersonal Violence Perpetrator; ivvictim = Interpersonal Violence Victim; criminalprob = Recent Criminal Legal Problem; civilprob = Legal Problem Other; intpartprob = Intimate Partner Problem; relaprob = Relationship Problem; alcoholprob = Alcohol Problem; subother = Substance Abuse Other; ampheta = Amphetamine Result; alcohol = Alcohol Result; opiate = Opiate Result; marijuana = Marijuana Result; cocaine = Cocaine Result; numbsub = Number of Substances; weapon = Weapon Type1.

© 2024 Xiao Y et al. *JAMA Psychiatry.*

eFigure 3. Three-Class Latent Class Solution

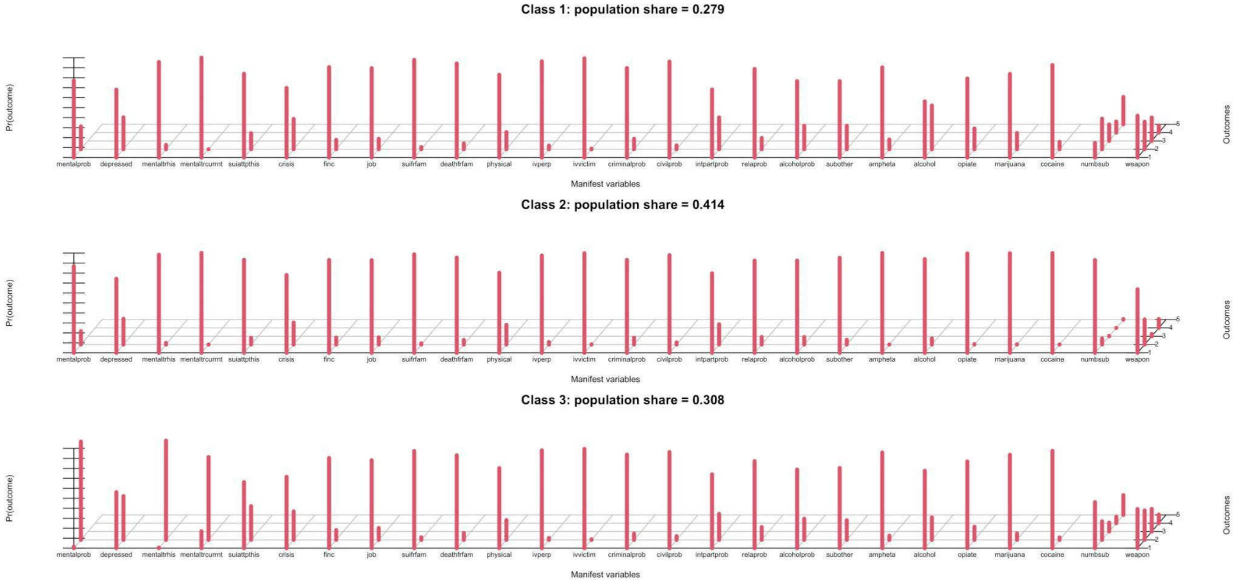

Note. mentalprob = Mental Health Problem; depressed = Depressed Mood; mentaltrhis = History Mental Illness Treatment; mentaltrcurrnt = Mental Illness Treatment Current; suiattpthis = Suicide Attempt History; crisis = Any Crisis; finc = Financial Problem; job = Job Problem; suifrfam = Recent Suicide Friend Family; deathfrfam = Death Friend Or Family Other; physical = Physical Health Problem; ivperp = Interpersonal Violence Perpetrator; ivvictim = Interpersonal Violence Victim; criminalprob = Recent Criminal Legal Problem; civilprob = Legal Problem Other; intpartprob = Intimate Partner Problem; relaprob = Relationship Problem; alcoholprob = Alcohol Problem; subother = Substance Abuse Other; ampheta = Amphetamine Result; alcohol = Alcohol Result; opiate = Opiate Result; marijuana = Marijuana Result; cocaine = Cocaine Result; numbsub = Number of Substances; weapon = Weapon Type1.

© 2024 Xiao Y et al. *JAMA Psychiatry.*

eFigure 4. Four-Class Latent Class Solution

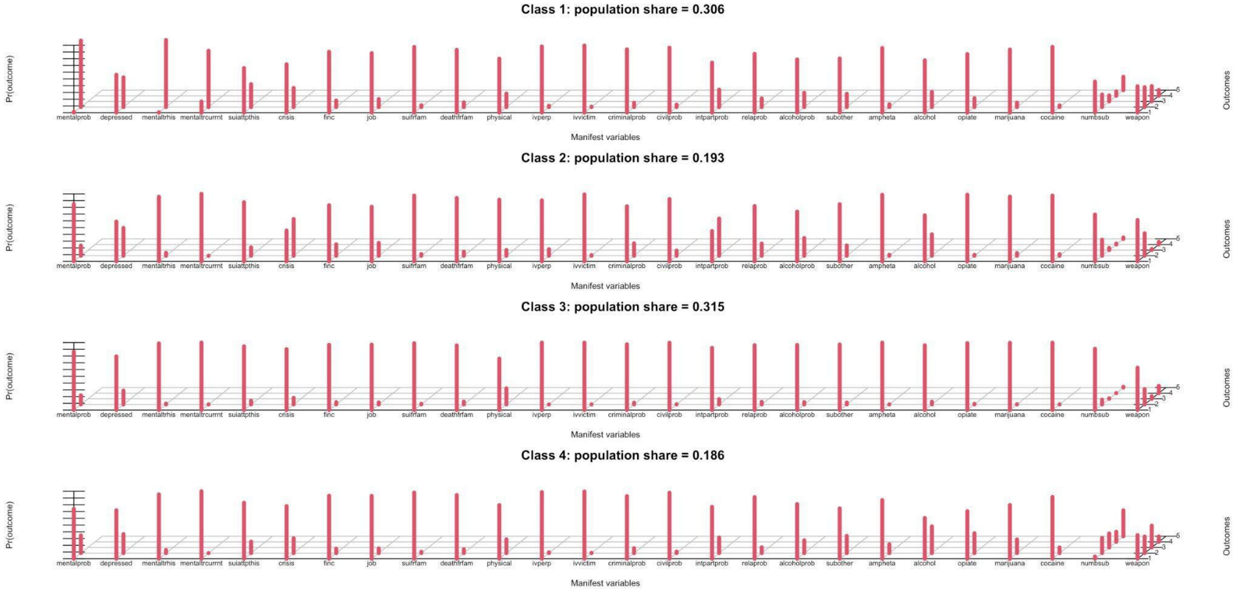

Note. mentalprob = Mental Health Problem; depressed = Depressed Mood; mentaltrhis = History Mental Illness Treatment; mentaltrcurrnt = Mental Illness Treatment Current; suiattpthis = Suicide Attempt History; crisis = Any Crisis; finc = Financial Problem; job = Job Problem; suifrfam = Recent Suicide Friend Family; deathfrfam = Death Friend Or Family Other; physical = Physical Health Problem; ivperp = Interpersonal Violence Perpetrator; ivvictim = Interpersonal Violence Victim; criminalprob = Recent Criminal Legal Problem; civilprob = Legal Problem Other; intpartprob = Intimate Partner Problem; relaprob = Relationship Problem; alcoholprob = Alcohol Problem; subother = Substance Abuse Other; ampheta = Amphetamine Result; alcohol = Alcohol Result; opiate = Opiate Result; marijuana = Marijuana Result; cocaine = Cocaine Result; numbsub = Number of Substances; weapon = Weapon Type1.

© 2024 Xiao Y et al. *JAMA Psychiatry.*

eFigure 5. Five-Class Latent Class Solution

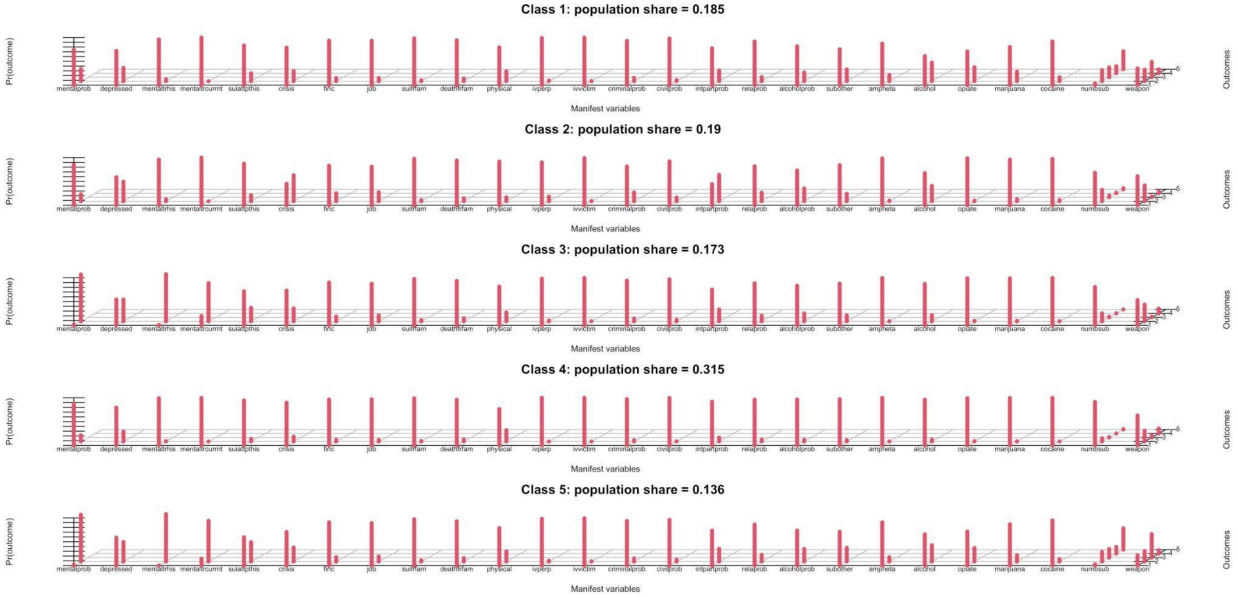

Note. mentalprob = Mental Health Problem; depressed = Depressed Mood; mentaltrhis = History Mental Illness Treatment; mentaltrcurrnt = Mental Illness Treatment Current; suiattpthis = Suicide Attempt History; crisis = Any Crisis; finc = Financial Problem; job = Job Problem; suifrfam = Recent Suicide Friend Family; deathfrfam = Death Friend Or Family Other; physical = Physical Health Problem; ivperp = Interpersonal Violence Perpetrator; ivvictim = Interpersonal Violence Victim; criminalprob = Recent Criminal Legal Problem; civilprob = Legal Problem Other; intpartprob = Intimate Partner Problem; relaprob = Relationship Problem; alcoholprob = Alcohol Problem; subother = Substance Abuse Other; ampheta = Amphetamine Result; alcohol = Alcohol Result; opiate = Opiate Result; marijuana = Marijuana Result; cocaine = Cocaine Result; numbsub = Number of Substances; weapon = Weapon Type1.

© 2024 Xiao Y et al. *JAMA Psychiatry.*

eFigure 6. Six-Class Latent Class Solution

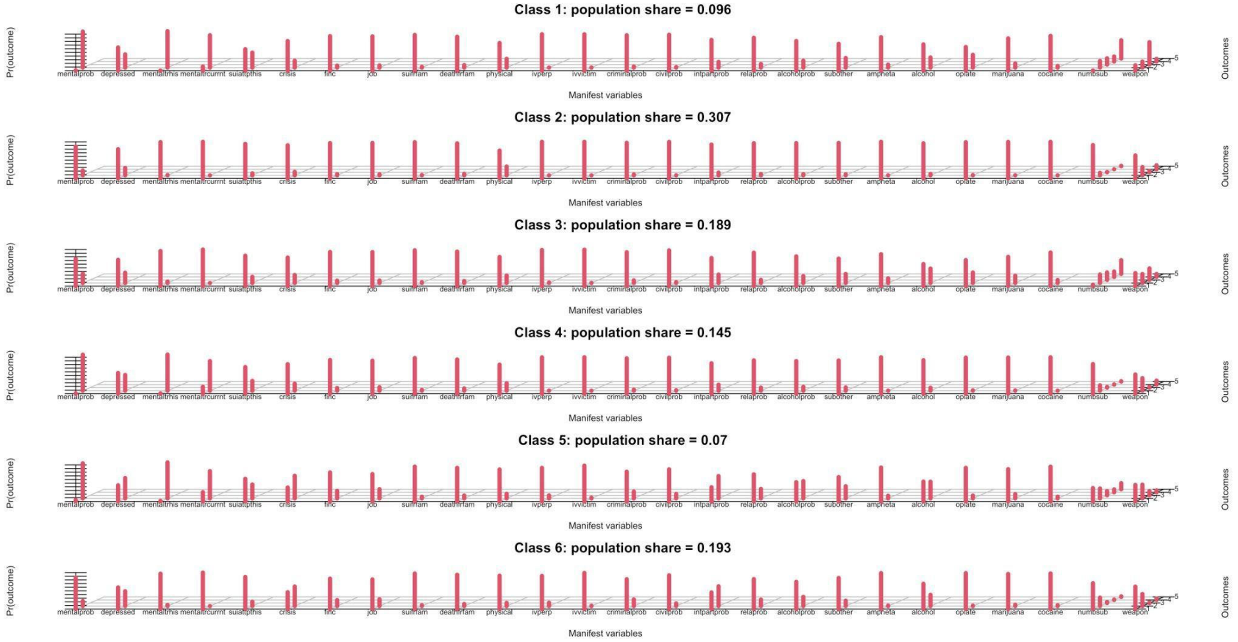

Note. mentalprob = Mental Health Problem; depressed = Depressed Mood; mentaltrhis = History Mental Illness Treatment; mentaltrcurrnt = Mental Illness Treatment Current; suiattpthis = Suicide Attempt History; crisis = Any Crisis; finc = Financial Problem; job = Job Problem; suifrfam = Recent Suicide Friend Family; deathfrfam = Death Friend Or Family Other; physical = Physical Health Problem; ivperp = Interpersonal Violence Perpetrator; ivvictim = Interpersonal Violence Victim; criminalprob = Recent Criminal Legal Problem; civilprob = Legal Problem Other; intpartprob = Intimate Partner Problem; relaprob = Relationship Problem; alcoholprob = Alcohol Problem; subother = Substance Abuse Other; ampheta = Amphetamine Result; alcohol = Alcohol Result; opiate = Opiate Result; marijuana = Marijuana Result; cocaine = Cocaine Result; numbsub = Number of Substances; weapon = Weapon Type1.

© 2024 Xiao Y et al. *JAMA Psychiatry.*

eFigure 7. Seven-Class Latent Class Solution

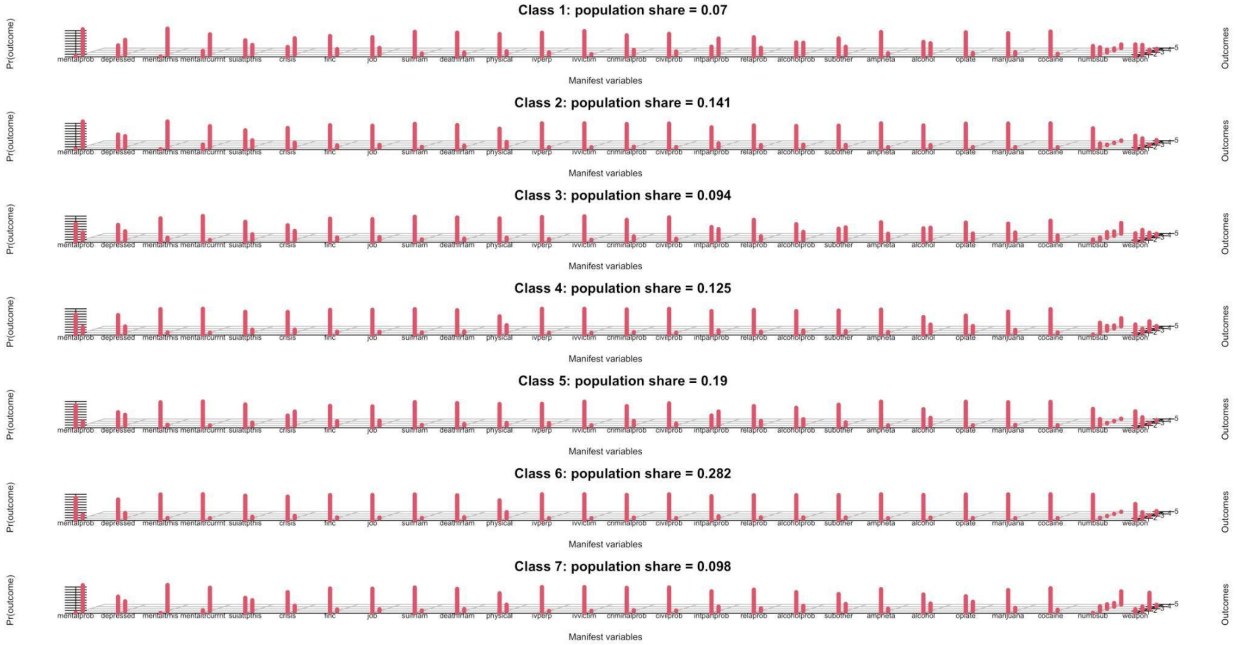

Note. mentalprob = Mental Health Problem; depressed = Depressed Mood; mentaltrhis = History Mental Illness Treatment; mentaltrcurrnt = Mental Illness Treatment Current; suiattpthis = Suicide Attempt History; crisis = Any Crisis; finc = Financial Problem; job = Job Problem; suifrfam = Recent Suicide Friend Family; deathfrfam = Death Friend Or Family Other; physical = Physical Health Problem; ivperp = Interpersonal Violence Perpetrator; ivvictim = Interpersonal Violence Victim; criminalprob = Recent Criminal Legal Problem; civilprob = Legal Problem Other; intpartprob = Intimate Partner Problem; relaprob = Relationship Problem; alcoholprob = Alcohol Problem; subother = Substance Abuse Other; ampheta = Amphetamine Result; alcohol = Alcohol Result; opiate = Opiate Result; marijuana = Marijuana Result; cocaine = Cocaine Result; numbsub = Number of Substances; weapon = Weapon Type1.

© 2024 Xiao Y et al. *JAMA Psychiatry.*

eFigure 8. Eight-Class Latent Class Solution

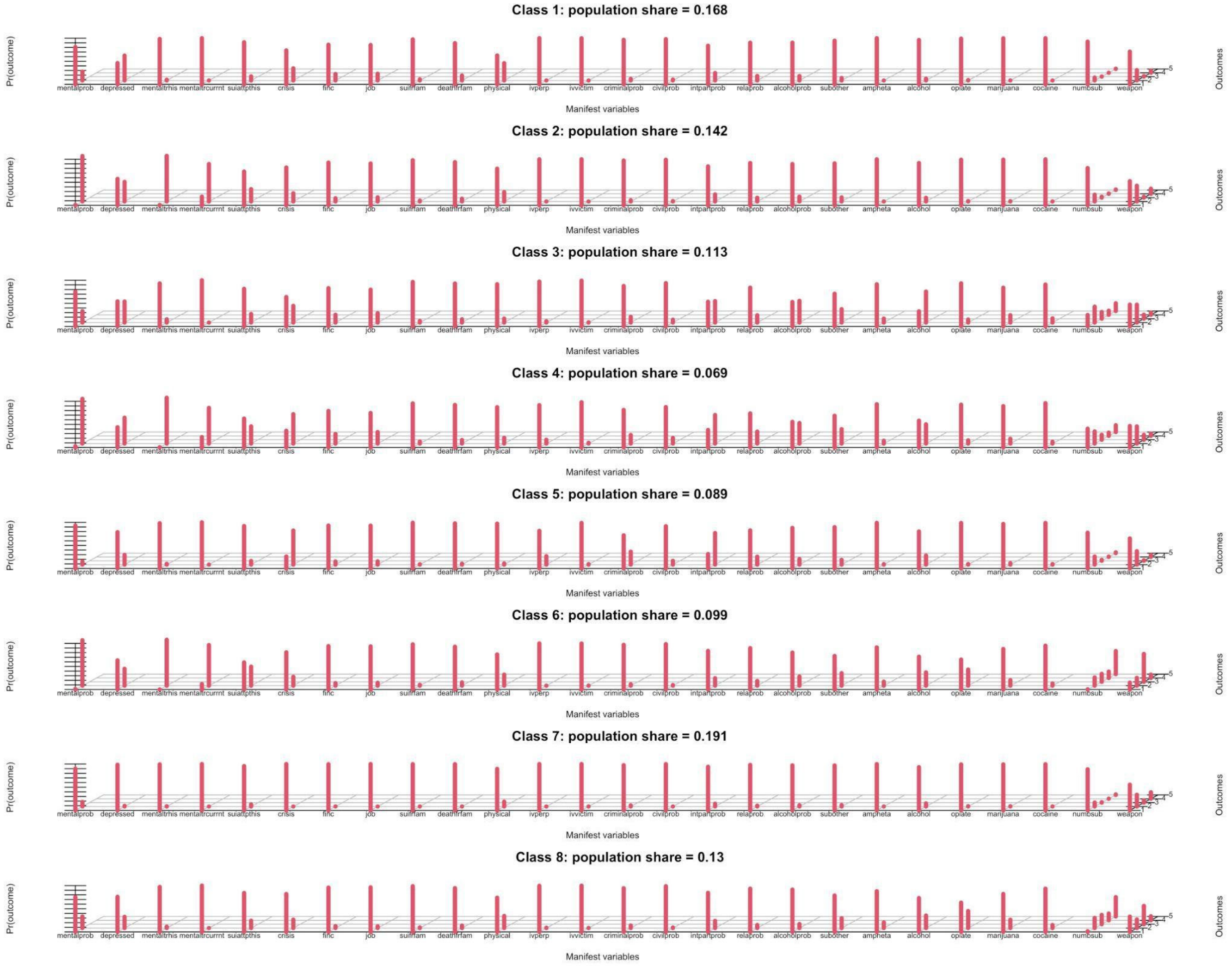

Note. mentalprob = Mental Health Problem; depressed = Depressed Mood; mentaltrhis = History Mental Illness Treatment; mentaltrcurrnt = Mental Illness Treatment Current; suiattpthis = Suicide Attempt History; crisis = Any Crisis; finc = Financial Problem; job = Job Problem; suifrfam = Recent Suicide Friend Family; deathfrfam = Death Friend Or Family Other; physical = Physical Health Problem; ivperp = Interpersonal Violence Perpetrator; ivvictim = Interpersonal Violence Victim; criminalprob = Recent Criminal Legal Problem; civilprob = Legal Problem Other; intpartprob = Intimate Partner Problem; relaprob = Relationship Problem; alcoholprob = Alcohol Problem; subother = Substance Abuse Other; ampheta = Amphetamine Result; alcohol = Alcohol Result; opiate = Opiate Result; marijuana = Marijuana Result; cocaine = Cocaine Result; numbsub = Number of Substances; weapon = Weapon Type1.

© 2024 Xiao Y et al. *JAMA Psychiatry.*

eFigure 9. Nine-Class Latent Class Solution

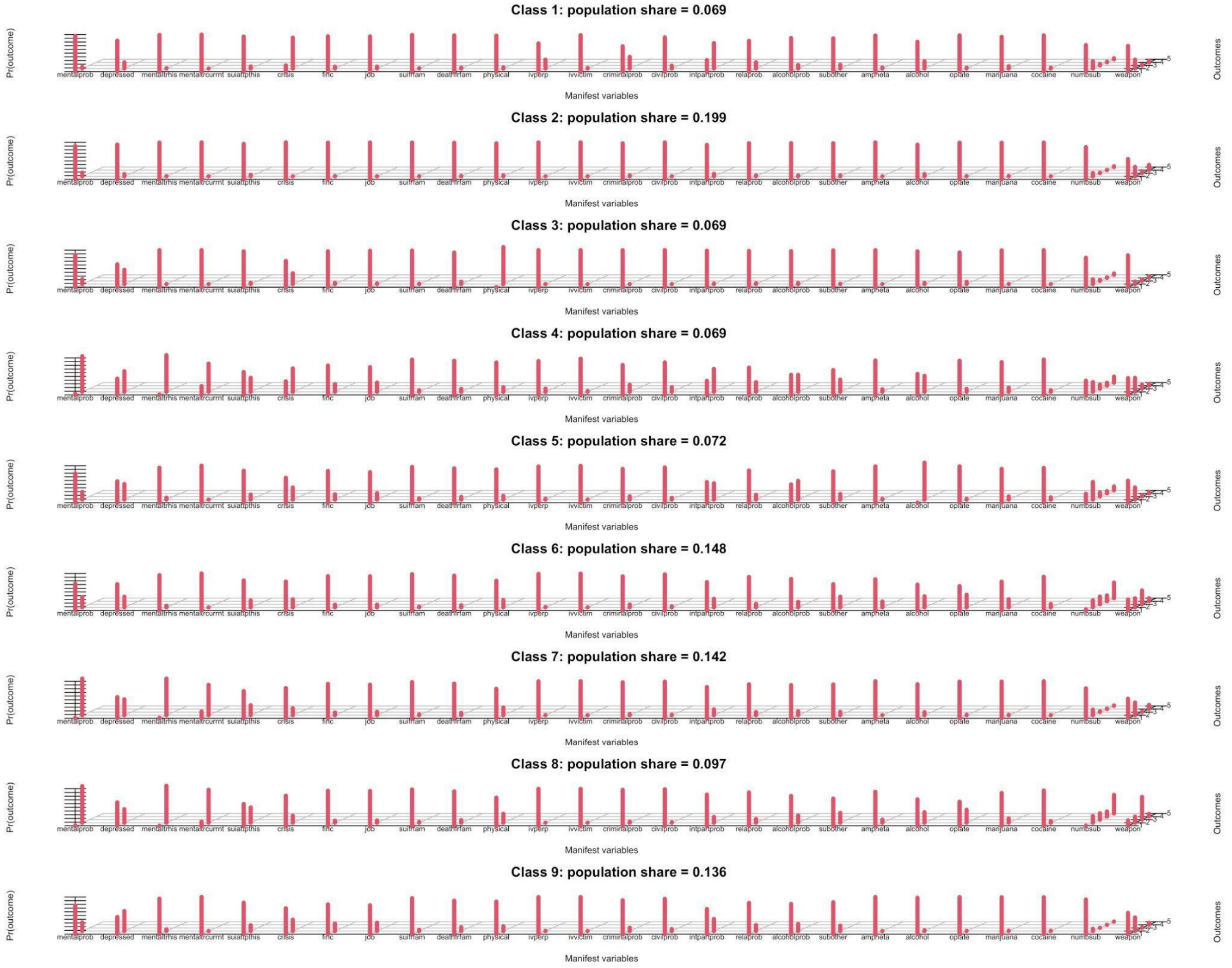

Note. mentalprob = Mental Health Problem; depressed = Depressed Mood; mentaltrhis = History Mental Illness Treatment; mentaltrcurrnt = Mental Illness Treatment Current; suiattpthis = Suicide Attempt History; crisis = Any Crisis; finc = Financial Problem; job = Job Problem; suifrfam = Recent Suicide Friend Family; deathfrfam = Death Friend Or Family Other; physical = Physical Health Problem; ivperp = Interpersonal Violence Perpetrator; ivvictim = Interpersonal Violence Victim; criminalprob = Recent Criminal Legal Problem; civilprob = Legal Problem Other; intpartprob = Intimate Partner Problem; relaprob = Relationship Problem; alcoholprob = Alcohol Problem; subother = Substance Abuse Other; ampheta = Amphetamine Result; alcohol = Alcohol Result; opiate = Opiate Result; marijuana = Marijuana Result; cocaine = Cocaine Result; numbsub = Number of Substances; weapon = Weapon Type1.

© 2024 Xiao Y et al. *JAMA Psychiatry.*

eFigure 10. Ten-Class Latent Class Solution

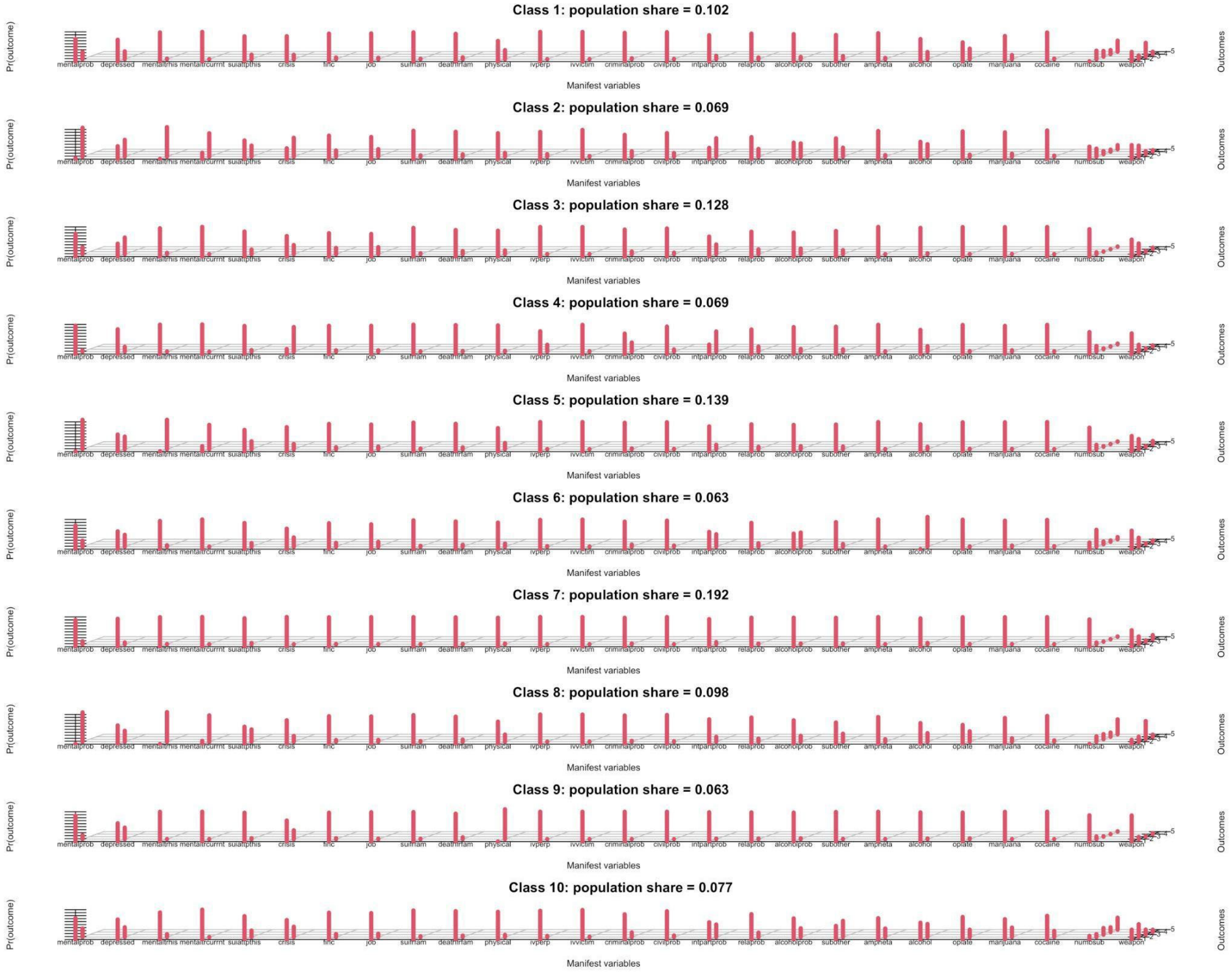

Note. mentalprob = Mental Health Problem; depressed = Depressed Mood; mentaltrhis = History Mental Illness Treatment; mentaltrcurrnt = Mental Illness Treatment Current; suiattpthis = Suicide Attempt History; crisis = Any Crisis; finc = Financial Problem; job = Job Problem; suifrfam = Recent Suicide Friend Family; deathfrfam = Death Friend Or Family Other; physical = Physical Health Problem; ivperp = Interpersonal Violence Perpetrator; ivvictim = Interpersonal Violence Victim; criminalprob = Recent Criminal Legal Problem; civilprob = Legal Problem Other; intpartprob = Intimate Partner Problem; relaprob = Relationship Problem; alcoholprob = Alcohol Problem; subother = Substance Abuse Other; ampheta = Amphetamine Result; alcohol = Alcohol Result; opiate = Opiate Result; marijuana = Marijuana Result; cocaine = Cocaine Result; numbsub = Number of Substances; weapon = Weapon Type1.

© 2024 Xiao Y et al. *JAMA Psychiatry.*

eTable 1. Indicator Information including Definition, Original Response Categories, and Recoding Rules

Indicator Name in Manuscript	Original Indicator Name in Dataset	Descriptions	Response Categories	Recoding Rules	Amount of Missing Values before Recoding	Amount of Missing Values after Recoding
**Indicators Used for Latent Class Analysis**						
mental health problem	mentalhealthproblem_c	Current mental health problem	0=No, Not Available, Unknown 1=Yes	N/A	0 (0.0%)	0 (0.0%)
depressed mood	depressedmood_c	Victim was perceived by self or others to be depressed at the time of the injury.	0=No, Not Available, Unknown 1=Yes	N/A	0 (0.0%)	0 (0.0%)
history of mental illness treatment	historymentalillnesstreatmnt_c	History of ever being treated for a mental health or substance abuse problem.	0=No, Not Available, Unknown 1=Yes	N/A	0 (0.0%)	0 (0.0%)
current mental illness treatment	mentalillnesstreatmentcurrnt_c	Currently in treatment for a mental health problem or substance abuse problem.	0=No, Not Available, Unknown 1=Yes	N/A	0 (0.0%)	0 (0.0%)
suicide attempt history	suicideattempthistory_c	Victim has a history of attempting suicide before the fatal incident.	0=No, Not Available, Unknown 1=Yes	N/A	0 (0.0%)	0 (0.0%)

© 2024 Xiao Y et al. *JAMA Psychiatry.*

recent crisis	anycrisis_c	Any crisis variable endorsed. Please note that in the analytic data file, the legacy data element “CrisisRecent” has been archived as of the 2016 dataset in order to avoid confusion. All cases endorsed as having a recent crisis in the legacy data, as well as current cases with a specific or “other” crisis as described above are captured under the calculated variable “AnyCrisis_c” in the analytic data file.	0=No, Not Available, Unknown 1=Yes	N/A	0 (0.0%)	0 (0.0%)
financial problem	financialproblem_c	Financial problems appear to have contributed to the death	0=No, Not Available, Unknown 1=Yes	N/A	0 (0.0%)	0 (0.0%)
job problem	jobproblem_c	Job problem(s) appear to have contributed to the death	0=No, Not Available, Unknown 1=Yes	N/A	0 (0.0%)	0 (0.0%)
recent suicide of friend family	recentsuicidefriendfamily_c	Suicide of a family member or friend appears to have contributed to the death.	0=No, Not Available, Unknown 1=Yes	N/A	0 (0.0%)	0 (0.0%)

© 2024 Xiao Y et al. *JAMA Psychiatry.*

death of friend or family	deathfriendorfamilyother_c	Death of a family member or friend due to something other than suicide appears to have contributed to the death.	0=No, Not Available, Unknown 1=Yes	N/A	0 (0.0%)	0 (0.0%)
physical health problem	physicalhealthproblem_c	Victim's physical health problem(s) appear to have contributed to the death. The victim was experiencing physical health problems (e.g., terminal disease, debilitating condition, chronic pain) that were relevant to the event.	0=No, Not Available, Unknown 1=Yes	N/A	0 (0.0%)	0 (0.0%)
perpetrator of interpersonal violence	interpersonalviolenceperp_c	Victim was a perpetrator of violence within the past month that was distinct and occurred before the violence that killed the victim.	0=No, Not Available, Unknown 1=Yes	N/A	0 (0.0%)	0 (0.0%)
victim of interpersonal violence	interpersonalviolencevictim_c	Victim experienced violence in the past month that was distinct and occurred before the violence that killed the victim.	0=No, Not Available, Unknown 1=Yes	N/A	0 (0.0%)	0 (0.0%)
criminal legal problem	recentcriminallegalproblem_c	Criminal legal problem(s) appear to have contributed to the death.	0=No, Not Available, Unknown 1=Yes	N/A	0 (0.0%)	0 (0.0%)

© 2024 Xiao Y et al. *JAMA Psychiatry.*

civil legal problem	legalproblemother_c	Civil legal (non-criminal) problem(s) appear to have contributed to the death	0=No, Not Available, Unknown 1=Yes	N/A	0 (0.0%)	0 (0.0%)
intimate partner problem	intimatepartnerproblem_c	Problems with a current or former intimate partner appear to have contributed to the suicide or undetermined death.	0=No, Not Available, Unknown 1=Yes	N/A	0 (0.0%)	0 (0.0%)
other relationship problem	relationshipproblemother_c+familyrelationship_c	Problems with a friend or associate (other than an intimate partner or family member) appear to have contributed to the death + Victim had relationship problems with a family member (other than an intimate partner) that appear to have contributed to the death.	0=No, Not Available, Unknown 1=Yes	N/A	0 (0.0%)	0 (0.0%)
alcohol problem	alcoholproblem_c	Person has alcohol dependence or alcohol problem.	0=No, Not Available, Unknown 1=Yes	N/A	0 (0.0%)	0 (0.0%)
other substance abuse	substanceabuseother_c	Person has a non-alcohol related substance abuse problem.	0=No, Not Available, Unknown 1=Yes	N/A	0 (0.0%)	0 (0.0%)

© 2024 Xiao Y et al. *JAMA Psychiatry.*

amphetamine test	amphetamineresult	Summary of substance results. Response Options: (1) Present; (2) Not present; (8) Not applicable (e.g., Testing was not done); and (9) Unknown	1=Present<br>2=Not Present<br>8=Not Applicable<br>9=Unknown	0=Not Present, Not Applicable, Unknown, NAs (blank cells)<br>1=Present	118 018 (38.5%)	0 (0.0%)
alcohol test	alcoholresult	Summary of substance results. Response Options: (1) Present; (2) Not present; (8) Not applicable (e.g., Testing was not done); and (9) Unknown	1=Present<br>2=Not Present<br>8=Not Applicable<br>9=Unknown	0=Not Present, Not Applicable, Unknown, NAs (blank cells)<br>1=Present	103 492 (33.7%)	0 (0.0%)
opiate test	opiateresult	Summary of substance results. Response Options: (1) Present; (2) Not present; (8) Not applicable (e.g., Testing was not done); and (9) Unknown	1=Present<br>2=Not Present<br>8=Not Applicable<br>9=Unknown	0=Not Present, Not Applicable, Unknown, NAs (blank cells)<br>1=Present	113 819 (37.1%)	0 (0.0%)

© 2024 Xiao Y et al. *JAMA Psychiatry.*

marijuana test	marijuanaresult	Summary of substance results. Response Options: (1) Present; (2) Not present; (8) Not applicable (e.g., Testing was not done); and (9) Unknown	1=Present<br>2=Not Present<br>8=Not Applicable<br>9=Unknown	0=Not Present, Not Applicable, Unknown, NAs (blank cells)<br>1=Present	118 586 (38.7%)	0 (0.0%)
cocaine test	cocaineresult	Summary of substance results. Response Options: (1) Present; (2) Not present; (8) Not applicable (e.g., Testing was not done); and (9) Unknown	1=Present<br>2=Not Present<br>8=Not Applicable<br>9=Unknown	0=Not Present, Not Applicable, Unknown, NAs (blank cells)<br>1=Present	118 097 (38.5%)	0 (0.0%)

© 2024 Xiao Y et al. *JAMA Psychiatry.*

number of substances	numbersubstances_c	Total number of substances for this victim at the time of death	Ranging from 1 to 66 (numeric)	1=None Present/NAs (blank cells)<br>2=1 type of substance<br>3=2 types of substance<br>4=3 types of substance<br>5=4 or more types of substance	174 001 (56.7%)	0 (0.0%)

© 2024 Xiao Y et al. *JAMA Psychiatry.*

Method type	Methodtype1	Type of Method or means used to inflict the fatal injury	1=Firearm<br>5=Non-powder gun<br>6=Sharp instrument<br>7=Blunt instrument<br>8=Poisoning<br>9=Hanging, strangulation, suffocation<br>10=Personal Methods<br>11=Fall<br>12=Explosive<br>13=Drowning<br>14=Fire or burns<br>15=Shaking, eg, shaken baby syndrome<br>16=Motor vehicle including buses, motorcycles<br>17=Other transport vehicle, eg, trains, planes, boats<br>18=Intentional neglect, eg, starving a baby<br>19=Biological Methods<br>66=Other (e.g. taser, electrocution, nail gun)<br>99=Unknown	1=Firearms<br>2=Hanging/Suffocation/Strangulation<br>3=Poisoning<br>4=All Other Weapon Types/Unknown/NAs (blank cells)	2365 (0.8%)	0 (0.0%)

© 2024 Xiao Y et al. *JAMA Psychiatry.*

Demographic Characteristics						
Age	Age	Age of victim (must use with AgeUnit)	Numeric age	<= 10 years old = Age group 1<br>11-15 years old = Age group 2<br>16-24 years old = Age group 3<br>25-40 years old = Age group 4<br>41-54 years old = Age group 5<br>55-70 years old = Age group 6<br>>=71 years old = Age group 7<br>999 recoded as missing	22 (0.01%)	175 (0.1%)

© 2024 Xiao Y et al. *JAMA Psychiatry.*

Sex	Sex	Biological sex of the victim	1=Male<br>2=Female<br>9=Unknown	Unknown recoded as missing	11 (0.004%)	65 (0.02%)
Educational Level	EducationLevel	Victim's educational level as measured by the highest degree attained or level completed	0=8th grade or less<br>1=9th to 12th grade, no diploma<br>2=High school graduate or GED completed<br>3=Some college credit, but no degree<br>4=Associate's degree<br>5=Bachelor's degree<br>6=Master's degree<br>7=Doctorate or Professional degree<br>9=Unknown	Unknown recoded as missing	5571 (1.8%)	59 324 (19.3%)
Race and Ethnicity	RaceEthnicity_c	Race and ethnicity of victim (combined)	1=White, Non-Hispanic<br>2=Black or African American, non-Hispanic<br>3=American Indian or Alaska Native, non-Hispanic<br>4=Asian/Pacific Islander, non-Hispanic<br>5=Other/Unspecified, non-Hispanic	Unknown recoded as missing	0 (0.0%)	322 (0.1%)

© 2024 Xiao Y et al. *JAMA Psychiatry.*

			6=Two or more races, non-Hispanic<br>7=Hispanic<br>9=Unknown race, Non-Hispanic			
Marital Status	MaritalStatus	Marital status of the victim (Domestic Partnership added in March 2015)	1=Married/Civil Union/Domestic Partnership<br>2=Never Married<br>3=Widowed<br>4=Divorced<br>5=Married/Civil Union/Domestic Partnership, but separated<br>6=Single, not otherwise specified<br>9=Unknown	Unknown recoded as missing	294 (0.1%)	3683 (1.2%)
Military Status	Military	Has the person ever served in the U.S. Armed Forces	0=No<br>1=Yes<br>9=Unknown	Unknown recoded as missing	934 (0.3%)	16 431 (5.4%)
Rural/Urban Residence	ResidenceFIPS for coding rural/urban areas	Residential county and state address of victim (5-digit FIPS code); rural-urban continuum code (RUCC)	Using rural-urban continuum code (RUCC) to categorize victim's residence to nonmetropolitan and metropolitan ares	1=Nonmetropolitan<br>2=Metropolitan	1585 (0.5%)	1615 (0.5%)

© 2024 Xiao Y et al. *JAMA Psychiatry.*

Outcomes (Warning Signs)						
Suicidal intent	SuicideIntentDisclosed_c	Victim disclosed to another person their thoughts and/or plans to commit suicide within the last month	0=No, Not Available, Unknown<br>1=Yes	N/A	0 (0.0%)	0 (0.0%)
Suicide notes	SuicideNote_c	Victim left a suicide note (or other recorded communication)	0=No, Not Available, Unknown<br>1=Yes	N/A	0 (0.0%)	0 (0.0%)
Psychotropic medications						0 (0.0%)
	AntiDepressantTested	Tested for Antidepressants	1=Tested<br>2=Not tested<br>9=Unknown	N/A	112 553 (36.7%)	112 553 (36.7%)
Antidepressant	AntiDepressantResult	Antidepressants results summary	1=Present<br>2=Not present<br>8=Not Applicable<br>9=Unknown	if AntiDepressantTested = 2 (Not tested), recode as 3 (Not tested)<br>If AntiDepressantResult = 8 or 9, recode as missing	119 543 (39.0%)	112 553 (42.2%)
Antipsychotic	AntipsychoticTested	Tested for Antipsychotics (added in August 2013)	1=Tested<br>2=Not tested<br>9=Unknown	N/A	197 186 (64.3%)	197 186 (64.3%)

© 2024 Xiao Y et al. *JAMA Psychiatry.*

	AntipsychoticResult	Antipsychotics results summary (added in August 2013)	1=Present<br>2=Not present<br>8=Not Applicable<br>9=Unknown	if AntipsychoticTested = 2 (Not tested), recode as 3 (Not tested)<br>If AntipsychotictResult = 8 or 9, recode as missing	205 304 (66.9%)	211 411 (68.9%)
	AnticonvulsantsTested	Tested for Anticonvulsants (added in August 2013)	1=Tested<br>2=Not tested<br>9=Unknown	N/A	196 367 (64.0%)	196 367 (64.0%)
Anticonvulsants	AnticonvulsantsResult	Anticonvulsants results summary (added in August 2013)	1=Present<br>2=Not present<br>8=Not Applicable<br>9=Unknown	if Anticonvulsantsested = 2 (Not tested), recode as 3 (Not tested)<br>If AnticonvulstansResult = 8 or 9, recode as missing	204 825 (66.8%)	211 042 (68.8%)

© 2024 Xiao Y et al. *JAMA Psychiatry.*

	BarbituratesTested	Tested for barbiturates (added in August 2013)	1=Tested<br>2=Not tested<br>9=Unknown	N/A	190 340 (62.0%)	190 340 (62.0%)
Barbiturates	BarbituratesResult	Barbiturates results summary (added in August 2013)	1=Present<br>2=Not present<br>8=Not Applicable<br>9=Unknown	if BarbituratesTested = 2 (Not tested), recode as 3 (Not tested)<br>If BarbituratesResult = 8 or 9, recode as missing	194 150 (63.3%)	198 801 (64.8%)

[a] Missing value in this set of variables was recoded into the "Not Applicable/Not present/Unknown" category.
[b] Missing value was recoded into "Others/Unknown" category for this variable (weapon type).
[c] Missing value was recoded as "Not Applicable/Not present/Unknown" category for this variable (number of substances present).

© 2024 Xiao Y et al. *JAMA Psychiatry.*

eTable 2. Model Fit Statistics of Latent Class Analysis Models.

Model Fit Statistics						Predicted Probability of Membership									
No. latent class	AIC	BIC	G^2	Entropy	Log Likelihood	1	2	3	4	5	6	7	8	9	10
1	6808201	6808531	1428250	NA	-3404070	1.00									
2	6316167	6316837	936151.2	0.97	-3158020	0.69	0.31								
3	6177844	6178854	797764.2	0.87	-30888827	0.42	0.27	0.31							
4	6114171	6115521	734027.2	0.83	-3056958	0.32	0.18	0.32	0.19						
**5**	**6052139**	**6053829**	**671930.9**	**0.83**	**-3025910**	**0.19**	**0.18**	**0.18**	**0.32**	**0.13**					
6	6025011	6027042	644739.5	0.81	-3012315	0.10	0.31	0.20	0.15	0.06	0.18				
7	6000661	6003032	620325.2	0.81	-3000107	0.06	0.14	0.09	0.14	0.18	0.29	0.10			
8	5980441	5983153	600041.8	0.77	-2989966	0.15	0.15	0.11	0.06	0.08	0.10	0.22	0.13		
9	5958913	5961965	578449.4	0.81	-2979170	0.07	0.20	0.08	0.06	0.08	0.15	0.15	0.10	0.12	
10	5939541	5942933	559013.5	0.81	-2969452	0.10	0.06	0.11	0.06	0.14	0.07	0.20	0.10	0.07	0.07

Note. AIC, Akaike Information Criterion. BIC, Bayesian Information Criterion. The bolded class was selected as the optimal model based on both interpretability and model fit statistics.

© 2024 Xiao Y et al. *JAMA Psychiatry.*

eTable 3. Distribution of Precipitating Circumstances by Five Suicide Decedent Profiles in the U.S., 2003-2020 (N=306 800)

Feature names	Class 1: Mental Health and Substance Problems	Class 2: Mental Health Problems	Class 3: Crisis, Alcohol-related, and Intimate Partner Problems	Class 4: Physical Health Problems	Class 5: Polysubstance Problems
**Mental Health Problem**	40979 (98.7%)	53305 (98.8%)	7655 (13.8%)	13033 (13.4%)	14421 (24.5%)
**Depressed Mood**	16981 (40.9%)	24556 (45.5%)	23132 (41.8%)	20710 (21.3%)	16068 (27.3%)
**History of Mental Illness Treatment**	41409 (99.7%)	53559 (99.3%)	2205 (4.0%)	668 (0.7%)	1697 (2.9%)
**Current Mental Illness Treatment**	35217 (84.8%)	43103 (79.9%)	5 (0.0%)	10 (0.0%)	7 (0.0%)
**Suicide Attempt History**	16811 (40.5%)	15134 (28.1%)	7119 (12.9%)	5561 (5.7%)	9617 (16.4%)
**Recent Crisis**	12206 (29.4%)	14776 (27.4%)	33474 (60.5%)	8354 (8.6%)	11899 (20.2%)
**Financial Problem**	3799 (9.1%)	5421 (10.1%)	9960 (18.0%)	3327 (3.4%)	3655 (6.2%)
**Job Problem**	4438 (10.7%)	6799 (12.6%)	11542 (20.8%)	2773 (2.9%)	3747 (6.4%)
**Recent Suicide of Friend/Family**	1139 (2.7%)	1373 (2.5%)	1446 (2.6%)	951 (1.0%)	1099 (1.9%)
**Death of Friend/Family**	3043 (7.3%)	3536 (6.6%)	3201 (5.8%)	4334 (4.5%)	3273 (5.6%)
**Physical Health Problem**	8816 (21.2%)	10111 (18.7%)	3718 (6.7%)	23647 (24.3%)	12201 (20.7%)
**Perpetrator of Interpersonal Violence**	737 (1.8%)	1064 (2.0%)	5845 (10.6%)	0 (0.0%)	528 (0.9%)
**Victim of Interpersonal Violence**	336 (0.8%)	180 (0.3%)	541 (1.0%)	19 (0.0%)	199 (0.3%)
**Criminal Legal Problem**	2748 (6.6%)	3254 (6.0%)	11489 (20.8%)	2116 (2.2%)	3872 (6.6%)
**Civil Legal Problem**	1657 (4.0%)	1818 (3.4%)	4721 (8.5%)	192 (0.2%)	1175 (2.0%)
**Intimate Partner Problem**	11066 (26.6%)	13633 (25.3%)	33499 (60.5%)	7024 (7.2%)	12422 (21.1%)
**Other Relationship Problem**	5820 (14.0%)	6318 (11.7%)	10771 (19.5%)	3707 (3.8%)	4675 (8.0%)
**Alcohol Problem**	10996 (26.5%)	9136 (16.9%)	15707 (28.4%)	3333 (3.4%)	10490 (17.8%)
**Other Substance Abuse**	12245 (29.5%)	6619 (12.3%)	9166 (16.6%)	2818 (2.9%)	13991 (23.8%)

© 2024 Xiao Y et al. *JAMA Psychiatry.*

**Amphetamine Test**	3982 (9.6%)	161 (0.3%)	740 (1.3%)	44 (0.0%)	7350 (12.5%)
**Alcohol Test**	14030 (33.8%)	7521 (13.9%)	18779 (33.9%)	2755 (2.8%)	23298 (39.6%)
**Opiate Test**	12047 (29.0%)	593 (1.1%)	636 (1.1%)	717 (0.7%)	16790 (28.6%)
**Marijuana Test**	5932 (14.3%)	497 (0.9%)	2236 (4.0%)	229 (0.2%)	11479 (19.5%)
**Cocaine Test**	2256 (5.4%)	244 (0.5%)	1530 (2.8%)	67 (0.1%)	4670 (7.9%)
**Number of Substance (0/Unknown)**	496 (1.2%)	43390 (80.5%)	38562 (69.6%)	90672 (93.3%)	881 (1.5%)
**Number of Substance (1)**	7564 (18.2%)	10111 (18.7%)	13817 (25.0%)	5569 (5.7%)	14344 (24.4%)
**Number of Substance (2)**	8186 (19.7%)	426 (0.8%)	1574 (2.8%)	394 (0.4%)	12404 (21.1%)
**Number of Substance (3)**	6252 (15.1%)	1 (0.0%)	619 (1.1%)	0 (0.0%)	8712 (14.8%)
**Number of Substance (4+)**	19029 (45.8%)	0 (0.0%)	795 (1.4%)	540 (0.6%)	22462 (38.2%)
**Method (Firearm)**	8518 (20.5%)	29030 (53.8%)	34149 (61.7%)	61674 (63.5%)	20220 (34.4%)
**Method (Hanging)**	9183 (22.1%)	18887 (35.0%)	18623 (33.6%)	22431 (23.1%)	14142 (24.0%)
**Method (Poisoning)**	20829 (50.2%)	751 (1.4%)	661 (1.2%)	2742 (2.8%)	19472 (33.1%)
**Method (Others)**	2997 (7.2%)	5260 (9.8%)	1934 (3.5%)	10328 (10.6%)	4969 (8.5%)

© 2024 Xiao Y et al. *JAMA Psychiatry.*

eTable 4. Results of Analysis without Missing Data Imputation

	Suicide Death without Warning	
	Non-disclosure of suicide intent	No Suicide note
	OR (95% CI)	OR (95% CI)
**Class (Ref: Class 1 - Mental health and substance problems)**		
Class 2 (Mental health problems)	1.06 (1.03-1.10)	1.14 (1.10-1.17)
Class 3 (Crisis and alcohol problems)	1.17 (1.13-1.21)	1.06 (1.03-1.10)
Class 4 (Physical health problems)	**2.62 (2.54-2.71)**	**1.44 (1.40-1.48)**
Class 5 (Polysubstance problems)	1.65 (1.60-1.71)	1.10 (1.07-1.14)
**Age**	1.00 (1.00-1.00)	1.00 (1.00-1.00)
**Sex (Ref: Male)**		
Female	1.07 (1.04-1.10)	0.72 (0.71-0.74)
**Education Level**	1.05 (1.05-1.06)	0.88 (0.88-0.89)
**Race/Ethnicity (Ref: White)**		
American Indian or Alaska Native, non-Hispanic	0.94 (0.87-1.02)	1.71 (1.56-1.87)
Asian/Pacific Islander, non-Hispanic	1.24 (1.16-1.33)	1.10 (1.04-1.17)
Black or African American, non-Hispanic	1.25 (1.20-1.30)	1.87 (1.79-1.95)
Hispanic	1.03 (0.99-1.07)	1.38 (1.33-1.43)
Other/Unspecified, non-Hispanic	1.48 (1.16-1.91)	1.25 (1.02-1.54)
Two or more races, non-Hispanic	0.82 (0.76-0.90)	0.98 (0.90-1.06)
**Marital Status (Ref: Married/Civil Union/Domestic Partnership)**		
Never Married	1.19 (1.16-1.23)	0.82 (0.80-0.84)

© 2024 Xiao Y et al. *JAMA Psychiatry.*

Widowed	0.94 (0.90-0.99)	0.78 (0.75-0.81)
Divorced	1.00 (0.97-1.02)	0.78 (0.76-0.80)
Married/Civil Union/Domestic Partnership, but separated	0.78 (0.73-0.82)	0.63 (0.60-0.67)
Single, not otherwise specified	1.05 (0.96-1.15)	0.85 (0.77-0.92)
**Military (Ref: No)**		
Yes	0.90 (0.88-0.93)	0.92 (0.90-0.95)
**Rural/urban residence (Ref: Rural)**		
Urban	1.00 (0.97-1.02)	0.83 (0.81-0.85)

Note: OR, odds ratio; CI, confidence interval; RUCC, Rural-Urban Continuum Codes.

© 2024 Xiao Y et al. *JAMA Psychiatry.*

eTable 5. Class Prevalence by Year

Year	Class Name	Number of Decedents in a Given Year	Number of Decedents by Year and Group	Percentage of Decedents by Year and Group
2003	Class 1 MH and Substance Problems	3623	396	10.93
2003	Class 2 MH Problems	3623	888	24.51
2003	Class 3 Crisis and Alcohol Problems	3623	800	22.08
2003	Class 4 Physical Health Problems	3623	1213	33.48
2003	Class 5 Polysubstance Problems	3623	326	9.00
2004	Class 1 MH and Substance Problems	7829	812	10.37
2004	Class 2 MH Problems	7829	1766	22.56
2004	Class 3 Crisis and Alcohol Problems	7829	2061	26.33
2004	Class 4 Physical Health Problems	7829	2460	31.42
2004	Class 5 Polysubstance Problems	7829	730	9.32
2005	Class 1 MH and Substance Problems	8976	959	10.68
2005	Class 2 MH Problems	8976	2055	22.89
2005	Class 3 Crisis and Alcohol Problems	8976	2307	25.70
2005	Class 4 Physical Health Problems	8976	2790	31.08
2005	Class 5 Polysubstance Problems	8976	865	9.64
2006	Class 1 MH and Substance Problems	9290	968	10.42
2006	Class 2 MH Problems	9290	2076	22.35
2006	Class 3 Crisis and Alcohol Problems	9290	2232	24.03
2006	Class 4 Physical Health Problems	9290	3090	33.26

© 2024 Xiao Y et al. *JAMA Psychiatry.*

2006	Class 5 Polysubstance Problems	9290	924	9.95
2007	Class 1 MH and Substance Problems	9683	1110	11.46
2007	Class 2 MH Problems	9683	2213	22.85
2007	Class 3 Crisis and Alcohol Problems	9683	2232	23.05
2007	Class 4 Physical Health Problems	9683	3197	33.02
2007	Class 5 Polysubstance Problems	9683	931	9.61
2008	Class 1 MH and Substance Problems	9895	1112	11.24
2008	Class 2 MH Problems	9895	2357	23.82
2008	Class 3 Crisis and Alcohol Problems	9895	2302	23.26
2008	Class 4 Physical Health Problems	9895	3222	32.56
2008	Class 5 Polysubstance Problems	9895	902	9.12
2009	Class 1 MH and Substance Problems	10329	1051	10.18
2009	Class 2 MH Problems	10329	2467	23.88
2009	Class 3 Crisis and Alcohol Problems	10329	2372	22.96
2009	Class 4 Physical Health Problems	10329	3536	34.23
2009	Class 5 Polysubstance Problems	10329	903	8.74
2010	Class 1 MH and Substance Problems	10646	1029	9.67
2010	Class 2 MH Problems	10646	2445	22.97
2010	Class 3 Crisis and Alcohol Problems	10646	2511	23.59
2010	Class 4 Physical Health Problems	10646	3766	35.37
2010	Class 5 Polysubstance Problems	10646	895	8.41

© 2024 Xiao Y et al. *JAMA Psychiatry.*

2011	Class 1 MH and Substance Problems	12442	1206	9.69
2011	Class 2 MH Problems	12442	3037	24.41
2011	Class 3 Crisis and Alcohol Problems	12442	2735	21.98
2011	Class 4 Physical Health Problems	12442	4356	35.01
2011	Class 5 Polysubstance Problems	12442	1108	8.91
2012	Class 1 MH and Substance Problems	12848	1686	13.12
2012	Class 2 MH Problems	12848	2764	21.51
2012	Class 3 Crisis and Alcohol Problems	12848	2443	19.01
2012	Class 4 Physical Health Problems	12848	4061	31.61
2012	Class 5 Polysubstance Problems	12848	1894	14.74
2013	Class 1 MH and Substance Problems	13100	1991	15.20
2013	Class 2 MH Problems	13100	2444	18.66
2013	Class 3 Crisis and Alcohol Problems	13100	2484	18.96
2013	Class 4 Physical Health Problems	13100	3877	29.60
2013	Class 5 Polysubstance Problems	13100	2304	17.59
2014	Class 1 MH and Substance Problems	14818	2007	13.54
2014	Class 2 MH Problems	14818	2577	17.39
2014	Class 3 Crisis and Alcohol Problems	14818	2779	18.75
2014	Class 4 Physical Health Problems	14818	4423	29.85
2014	Class 5 Polysubstance Problems	14818	3032	20.46
2015	Class 1 MH and Substance Problems	20561	3158	15.36

© 2024 Xiao Y et al. *JAMA Psychiatry.*

2015	Class 2 MH Problems	20561	3203	15.58
2015	Class 3 Crisis and Alcohol Problems	20561	3370	16.39
2015	Class 4 Physical Health Problems	20561	5755	27.99
2015	Class 5 Polysubstance Problems	20561	5075	24.68
2016	Class 1 MH and Substance Problems	25862	4157	16.07
2016	Class 2 MH Problems	25862	3952	15.28
2016	Class 3 Crisis and Alcohol Problems	25862	4220	16.32
2016	Class 4 Physical Health Problems	25862	7223	27.93
2016	Class 5 Polysubstance Problems	25862	6310	24.40
2017	Class 1 MH and Substance Problems	29765	4821	16.20
2017	Class 2 MH Problems	29765	4683	15.73
2017	Class 3 Crisis and Alcohol Problems	29765	4726	15.88
2017	Class 4 Physical Health Problems	29765	8572	28.80
2017	Class 5 Polysubstance Problems	29765	6963	23.39
2018	Class 1 MH and Substance Problems	35029	5138	14.67
2018	Class 2 MH Problems	35029	5373	15.34
2018	Class 3 Crisis and Alcohol Problems	35029	5371	15.33
2018	Class 4 Physical Health Problems	35029	10929	31.20
2018	Class 5 Polysubstance Problems	35029	8218	23.46
2019	Class 1 MH and Substance Problems	33355	4806	14.41
2019	Class 2 MH Problems	33355	4859	14.57

© 2024 Xiao Y et al. *JAMA Psychiatry.*

2019	Class 3 Crisis and Alcohol Problems	33355	4986	14.95
2019	Class 4 Physical Health Problems	33355	10507	31.50
2019	Class 5 Polysubstance Problems	33355	8197	24.58
2020	Class 1 MH and Substance Problems	38749	5120	13.21
2020	Class 2 MH Problems	38749	4769	12.31
2020	Class 3 Crisis and Alcohol Problems	38749	5436	14.03
2020	Class 4 Physical Health Problems	38749	14198	36.64
2020	Class 5 Polysubstance Problems	38749	9226	23.81

© 2024 Xiao Y et al. *JAMA Psychiatry.*

eTable 6. Results of Analysis without Missing Data Imputation Additionally controlling for Fixed Effects of Year and State

	Signs of Suicide Intent	
	Non-disclosure of suicide intent	No Suicide note
	OR (95% CI)	OR (95% CI)
**Class (Ref: Class 1 - Mental health and substance problems)**		
Class 2 (Mental health problems)	1.12 (1.09-1.16)	1.13 (1.09-1.16)
Class 3 (Crisis and alcohol problems)	1.24 (1.20-1.28)	1.07 (1.03-1.10)
Class 4 (Physical health problems)	**2.48 (2.39.-2.56)**	**1.24 (1.18-1.25)**
Class 5 (Polysubstance problems)	1.57 (1.52-1.62)	1.02 (0.99-1.05)

Note: OR, odds ratio; CI, confidence interval.

© 2024 Xiao Y et al. *JAMA Psychiatry.*

eReferences

1. Meyer BD, Mittag N. Misclassification in binary choice models. *J Econom*. 2017;200(2):295-311.

2. Stefanski LA, Carroll RJ. Covariate Measurement Error in Logistic Regression. *Ann Stat*. 1985;13(4):1335-1351.

3. Liu GS, Nguyen BL, Lyons BH, et al. Surveillance for Violent Deaths - National Violent Death Reporting System, 48 States, the District of Columbia, and Puerto Rico, 2020. *MMWR Surveill Summ*. 2023;72(5):1-38.

© 2024 Xiao Y et al. *JAMA Psychiatry.*
