# Supplementary material for: Decoding Suicide Decedent Profiles and Signs of Suicidal Intent Using Latent Class Analysis
Source: JAMA Psychiatry. 2024 Mar 20;81(6):595–605. doi: 10.1001/jamapsychiatry.2024.0171 (PMC10955339; doi:10.1001/jamapsychiatry.2024.0171)
Supplement: Supplement 2. — Data sharing statement [file jamapsychiatry-e240171-s002.pdf]

Data Sharing Statement

Xiao. Decoding Suicide Decedent Profiles and Signs of Suicidal Intent Using Latent Class Analysis. *JAMA Psychiatry*. Published March 20, 2024. doi:10.1001/jamapsychiatry.2024.0171

Data
Data available: No

Additional Information
Explanation for why data not available: The Restricted Access Data file (RAD) from the National Violent Death Reporting System (NVDRS) can be obtained through a request to the Centers for Disease Control and Prevention (CDC).
